# Wide-field multiphoton imaging through scattering media without correction

**DOI:** 10.1126/sciadv.aau1338

**Published:** 2018-10-12

**Authors:** Adrià Escobet-Montalbán, Roman Spesyvtsev, Mingzhou Chen, Wardiya Afshar Saber, Melissa Andrews, C. Simon Herrington, Michael Mazilu, Kishan Dholakia

**Affiliations:** 1 SUPA, School of Physics and Astronomy, University of St. Andrews, North Haugh, St. Andrews KY16 9SS, UK.; 2 School of Medicine, University of St. Andrews, North Haugh, St. Andrews KY16 9FT, UK.; 3 Biological Sciences, University of Southampton, University Road, Southampton SO17 1BJ, UK.; 4 CRUK Edinburgh Centre, Institute of Genetics and Molecular Medicine, The University of Edinburgh, Crewe Road South, Edinburgh EH4 2XR, UK.

## Abstract

Optical approaches to fluorescent, spectroscopic, and morphological imaging have made exceptional advances in the last decade. Super-resolution imaging and wide-field multiphoton imaging are now underpinning major advances across the biomedical sciences. While the advances have been startling, the key unmet challenge to date in all forms of optical imaging is to penetrate deeper. A number of schemes implement aberration correction or the use of complex photonics to address this need. In contrast, we approach this challenge by implementing a scheme that requires no a priori information about the medium nor its properties. Exploiting temporal focusing and single-pixel detection in our innovative scheme, we obtain wide-field two-photon images through various turbid media including a scattering phantom and tissue reaching a depth of up to seven scattering mean free path lengths. Our results show that it competes favorably with standard point-scanning two-photon imaging, with up to a fivefold improvement in signal-to-background ratio while showing significantly lower photobleaching.

## Teaser

Focusing ultrashort laser pulses in time in tandem with single-pixel detection images at depth with no sample characterization.

## INTRODUCTION

A suite of powerful, disruptive optical imaging approaches across the physical and biomedical sciences has recently emerged. Super-resolution imaging led to new studies looking at nanometric features within cells that have revealed intricate aspects of subcellular processes (*1*–*5*). At the larger scale, methods such as optical coherence tomography (*6*) and light-sheet imaging (*7*) are taking hold in fields such as opthalmology, neuroscience, and developmental biology. In tandem with the requirement for a fast, wide-field visualization and super-resolved imaging across biomedicine, a grand challenge is to perform such imaging through highly scattering (turbid) media, namely, tissue. In particular, this is essential to move from superficial surface imaging to functional imaging at depth (*8*–*11*), which is crucial for biomedical areas including neuroscience. To address this area, aberration correction can be implemented (*12*). However, this does not readily take into account the properties of the medium, and actual retrieval of the emitted signal from depth in the medium can still be challenging. Key advances have emerged by a consideration of the propagation of light within a complex medium. In this field, a number of approaches use dynamic wavefront shaping for illumination of the sample with a calculated input complex wavefront (*13*–*16*), which can focus light upon an embedded guide star. In essence, one determines the transmission matrix of the sample in this process (*11*, *17*, *18*). While this is powerful, the requirement of a guide star restricts the approach. Furthermore, it requires determination of the properties of the medium at one or more individual points, making it very challenging to implement for wide-field imaging.

An important advance would be the realization of a fast, wide-field imaging approach that would deliver and retrieve light from any given plane within a sample, even in the presence of turbidity. This would be without the requirement to characterize or even actively correct the aberrating effect of the turbid medium. Our approach to achieving this goal exploits temporal focusing (TF) microscopy (*19*, *20*). By using the temporal rather than spatial degree of freedom, scanning of the optical axis for image reconstruction is avoided. Consequently, TF may record wide-field multiphoton images (*19*, *21*). In addition, a little-recognized facet of TF is its ability to deliver light through scattering media. This ability has been used to project optical patterns for applications such as optogenetics, providing photostimulation at remarkable depths (*9*, *22*, *23*). Although TF can deliver light through a scattering medium very efficiently, collecting the emitted fluorescent light back through the same medium (i.e., truly achieving imaging) has not been accomplished to date. Separately, there has been the emergence of single-pixel detection, sometimes termed computational ghost imaging (*24*). In this form of imaging, known patterns illuminate an object, a single-element photodetector records the light intensity that is either transmitted or backscattered by the object, and images are reconstructed with the appropriate algorithm (*25*, *26*).

However, while these studies in TF microscopy and in single-pixel detection have shown promise, none of them has addressed the challenge of correction-free wide-field imaging through turbid media. The scheme that we present here, which we call TempoRAl Focusing microscopy with single-pIXel detection (TRAFIX), uses a judicious combination of TF illumination with single-pixel imaging to obtain wide-field images of fluorescent microscopic samples within or even beyond biological tissues, in the presence of multiple scattering, without aberration correction or characterization of the turbid medium.

## RESULTS

### Principle of the technique

**Fig. 1:**
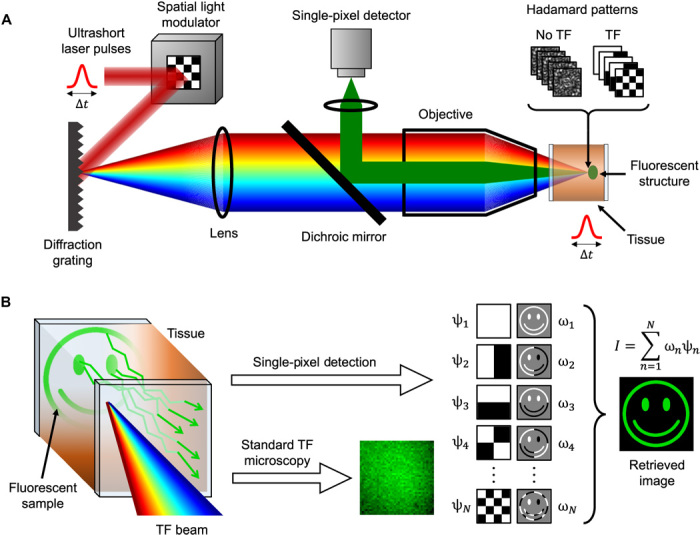
Working principle of TRAFIX. (**A**) A femtosecond laser beam is expanded onto a spatial light modulator (SLM) that generates Hadamard patterns. Subsequently, the beam is diffracted from a grating, and the Hadamard patterns are projected onto a fluorescent sample after propagating through a scattering medium. Fluorescent light emitted by the sample is collected by the same objective after passing through the scattering medium a second time (epifluorescence geometry), and the total intensity is measured by a single-pixel detector. (**B**) A TF beam propagates through a turbid medium with minimal distortion, retaining the integrity of illumination patterns in the sample plane. Emitted fluorescent photons scatter as they propagate back through the tissue. In contrast to standard TF microscopy, TRAFIX tolerates scrambling of back-propagating light since only an intensity measurement is performed. In a single-pixel measurement, the fluorescent target is sequentially illuminated with Hadamard patterns (ψ_*n*_), and the total intensity detected is stored as a coefficient (ω_*n*_). Gray background in the second column denotes regions of zero intensity. By adding up the Hadamard patterns weighted by their respective coefficients, an image of the fluorescent sample is reconstructed.

TF is based on decomposing an incident ultrashort pulsed light field into its constituent wavelengths with a diffraction grating. Each wavelength propagates along an individual path in the optical system, and these wavelengths constructively recombine to regain the original pulse duration only at the plane conjugate to the grating, generating axially confined mutiphoton excitation. In TRAFIX, orthonormal light patterns (in a Hadamard basis) are temporally focused through a turbid medium to illuminate a fluorescent microscopic sample of interest. The use of TF for this projection ensures the retention of the integrity of these patterns at any given plane within the turbid media (Fig. 1A). This can be regarded as due to the fact that ballistic photons remain unperturbed all the way to the object plane and arrive at the same time, contributing to the reconstitution of the pulse. In addition, the superposition of wavelets of slightly different wavelengths at the focal plane results in nearly speckle-free propagation through long distances in scattering media, as recognized by Papagiakoumou *et al*. (*9*). We confirm these aspects here with a numerical simulation. The same principle has been previously used for reducing out-of-focus excitation in line-scanning multiphoton microscopy (*20*). A scattering medium may affect the spatial and temporal degrees of freedom of an input field differently. In the time domain, the temporal profile of femtosecond pulses is not significantly distorted at substantial imaging depths such as 1-mm-thick brain tissue (*27*). As a consequence, TF may induce much more efficient multiphoton excitation when compared to standard point-focusing where spatial speckle greatly reduces the photon density at the focal spot. Consequently, TF is more robust than conventional focusing, resulting in a more intense fluorescence signal generated at large depths (*28*), which is a major attribute for our approach.

The total intensity emitted by the fluorescent sample under each illumination pattern is collected by the same objective after passing a second time through the scattering material, in a configuration reminiscent of a single-pixel imaging. In this way, we remove the requirement for any spatial resolution on the imaging path, which, in turn, means that we can readily tolerate the scrambling of the emitted fluorescence through the scattering medium (Fig. 1B). We retain exact spatial information of where the sample is illuminated by virtue of using patterned illumination. This allows an original form of TF microscopy to be realized, enabling the use of the full penetration capabilities of TF beams for imaging at depth (*29*).

In the present experiments, an illumination laser with a central wavelength at 800 nm delivers 140-fs pulses (80-MHz repetition rate, average output power up to 4 W) onto the sample, and the emitted fluorescent photons are detected by an electron-multiplying charge-coupled device (EMCCD) camera, which is used as a single-pixel detector. The epifluorescence configuration of TRAFIX makes it readily suitable for a suite of biomedical applications. An additional microscope takes reference images of the fluorescent sample in a transmission geometry analogous to previous reports (*9*, *22*). Reference images are taken with a CCD camera under uniform TF illumination across the field of view (FOV) (see Materials and Methods). We stress that this additional reference system is not required for imaging. Once all patterns have been sequentially projected and their intensity coefficients have been measured, images are reconstructed using an orthogonal matching pursuit (OMP) algorithm (*30*). The OMP algorithm determines which patterns contribute most effectively to the image reconstruction and sums them up to create an image (see the Supplementary Materials). The number of pixels in the retrieved image is determined by the size of the Hadamard basis used in the measurement. An *n* × *n* pixel image requires a Hadamard basis containing *N* = *n*^2^ patterns. Therefore, depending on the pixel resolution required, a different number of Hadamard patterns (typically 4096 or 1024) are encoded on an SLM. The acquisition time of the microscope is given by *T* = 2*n*^2^(*t*_exp_ + *t*_SLM_), where *t*_exp_ is the exposure time of the camera used as a single-pixel detector and *t*_SLM_ is the time required to refresh the Hadamard patterns on the SLM (including data transmission).

As TRAFIX is based on patterned illumination, it lends itself to compressive sensing measurements (*26*, *31*). One of the main advantages of compressive sensing is that sparse signals can be reconstructed with fewer samples than required by Nyquist sampling theory. In terms of microscopy, it means that one does not need to measure with the full set of Hadamard patterns to obtain a good-quality image. The compression ratio (CR) = *N*/*M* denotes how many patterns are used to reconstruct the image in relation to the total number of patterns in the Hadamard basis (*26*). Here, *M* is the number of patterns used in the reconstruction algorithm. For example, a 64-pixel by 64-pixel image requires a measurement with a Hadamard basis containing 4096 patterns. Consequently, a CR of 2 corresponds to using only half of the total patterns to reconstruct the image (i.e., 2048 patterns), a CR of 4 uses only 1024 patterns, and so on.

As we describe below, to demonstrate the performance of TRAFIX, we imaged various microscopic fluorescent samples making use of full Hadamard bases to obtain a high-quality image. Additional compressed images were obtained a posteriori to demonstrate that compressive sensing measurements are possible in this configuration (see the Supplementary Materials).

### Imaging through scattering media

To begin with, 400-nm-diameter green fluorescent beads and fixed human embryonic kidney (HEK) 293T/17 cells labeled with green fluorescent protein (HEK293T/17-GFP) were imaged through scattering phantoms, designed to mimic the scattering properties of biological tissue, and through unfixed human colon tissue. A custom-made fluorescent microstructure was then imaged through fixed rat brain tissue. As scattering dominates over absorption in the range of wavelengths considered in our investigation (*32*, *33*), we use scattering mean free path (*l*_s_) as a reference value to quantify imaging depth. The *l*_s_ values for the scattering media used in the experiments presented here are approximately 140, 85, and 55 μm for the scattering phantom, colon tissue, and brain tissue, respectively (see Materials and Methods). Full Hadamard bases of either 32-pixel by 32-pixel or 64-pixel by 64-pixel resolution were projected onto the samples, and the resulting images were reconstructed with different CRs. The lateral resolution of the microscope is defined as twice the pixel size in the reconstructed images, and thus, it depends on the FOV. Using a Hadamard basis containing 32 pixels by 32 pixels and an FOV of 90 μm by 90 μm, the resolution is 5.6 μm, and for a larger basis of 64 pixels by 64 pixels, it is 2.8 μm (fig. S7). We measured the depth resolution in the absence of any scattering layer for a 40× numerical aperture (NA) = 0.8 objective to be 4.7 ± 0.5 μm (fig. S4) and initially tested the microscope’s performance in imaging beads and HEK cells without scattering (see the Supplementary Materials).

To quantify image quality, we measured the signal-to-background ratio (SBR) for all images presented in this work (see Materials and Methods). All values are summarized in table S1. To assess the effectiveness of this technique in photolocalization, we estimated the spacing between fluorescent beads and the size of cells in the reference image and in the reconstructed images and calculated their deviation from the reference value (see the Supplementary Materials).

**Fig. 2:**
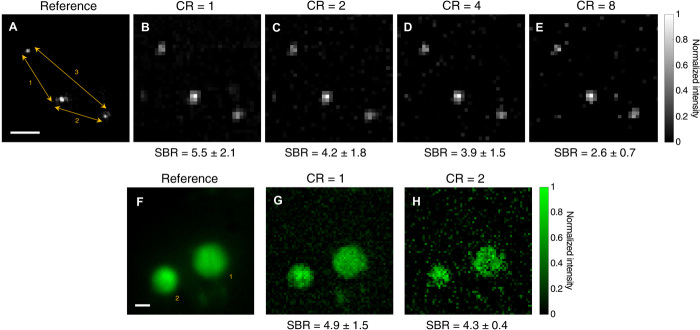
Images of fluorescent microscopic samples through scattering phantoms. Fluorescent beads of 400 nm in diameter and fixed HEK293T/17-GFP cells were imaged through 500- and 540-μm of scattering phantoms, respectively. (**A** and **F**) Images taken from the reference imaging system under uniform TF illumination across the FOV. Exposure time was set to 20 s, and camera binning was 4 × 4 (beads) and 2 × 2 (cells). (**B** to **E**, **G**, and **H**) Images obtained in epifluorescence configuration with TRAFIX using a Hadamard basis containing 4096 illumination patterns. They were reconstructed with different CRs corresponding to 100% (CR = 1), 50% (CR = 2), 25% (CR = 4), or 12.5% (CR = 8) of the total patterns. Each measurement under individual illumination patterns was taken with a binning of 64 × 64 and an exposure time of 0.5 s. The spacing between beads was measured in all five images obtaining deviations smaller than 3% from the reference image (table S3). The diameters of the cells in (F) were measured to be 20.7 and 14.3 μm, respectively, and their values in (G) and (H) differ less than 4 and 12% from the reference value (table S2). The SBR is shown for all reconstructed images. Scale bars, 10 μm.

Figure 2 shows images of the fluorescent beads and HEK cells obtained through 500 and 540 μm of scattering phantoms (3 to 4 *l*_s_), respectively. As a result of the strong scattering, very few illumination photons reach the sample plane, generating a very dim fluorescence signal; therefore, a long exposure time or large binning is required to even obtain a good reference image under uniform TF illumination in transmission. In standard TF microscopes, those few emitted fluorescence photons would need to travel back through the thick scattering medium, losing all their spatial information and hence resulting in uniform noise on the camera (*29*). Consequently, the SBR would essentially be unity, which means that no signal can be extracted from the background noise. However, in TRAFIX, there is no need to measure spatial information as total intensity coming from the sample under each illumination pattern is detected and used as a coefficient in the reconstruction algorithm. This results in a significant enhancement in signal detection and higher SBR values.

The achievable level of compression depends on the sparsity of the image. If an image contains very little information, then high compression is possible with negligible information loss. As the images of fluorescent beads are sparse, image quality is preserved for large values of CR. A good image can be faithfully reconstructed with CR = 8 (12.5% of the total number of patterns). Similar CRs have been achieved in the literature, although with different optical imaging systems (*26*). Nevertheless, we did not achieve a high compression with cells in the present configuration. They covered a larger area of the image and thus cannot be compressed as much without a significant loss of information with the present algorithm and pixel resolution. In addition, the 800-nm excitation laser used in the experiments is not optimal for excitation of GFP, which has its highest absorption in the range of wavelengths between 870 and 920 nm (*34*). As a result, the cells appear very dim. Despite these issues, an SBR exceeding 4 was achieved at CR = 2 (i.e., using only 50% of the full Hadamard basis in the image reconstruction algorithm).

**Fig. 3:**
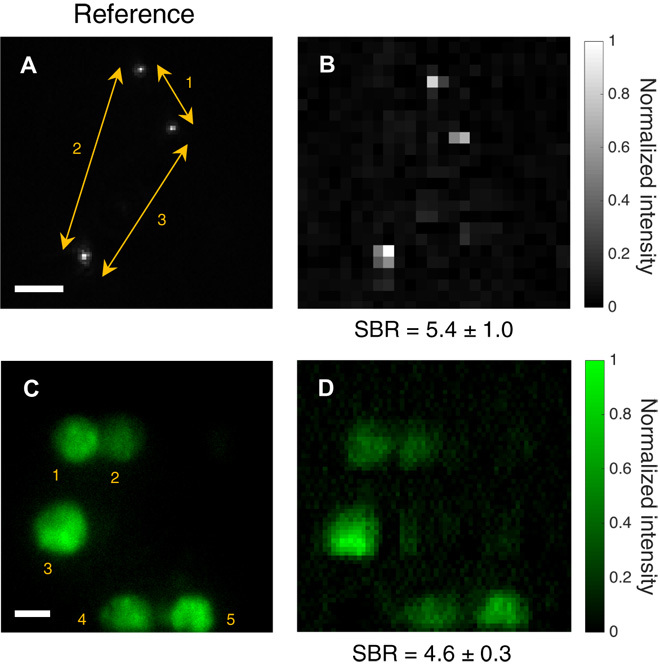
Images of fluorescent microscopic samples through unfixed human colon tissue. Fluorescent beads of 400 nm in diameter and fixed HEK293T/17-GFP cells were imaged through 250 and 200 μm of human colon tissue, respectively. (**A** and **C**) Images taken from the reference imaging system under uniform TF illumination across the FOV. Camera binning in (A) was set to 4 × 4, and exposure time was 5 s. No camera binning was used in (C), and exposure time was 15 s. (**B** and **D**) Images obtained with TRAFIX using a Hadamard basis containing 1024 and 4096 illumination patterns, respectively. All patterns were used for image reconstruction (CR = 1). Camera binning for each Hadamard pattern was set to 64 × 64, and exposure time values were (B) 1 s and (D) 0.75 s. The spacing between beads and the diameter of cells were measured to assess image quality (tables S3 and S2, respectively). The SBR is shown for all reconstructed images. Scale bars, 10 μm.

In the next stage, the performance of TRAFIX was tested with unfixed human colon tissue as a scattering medium. One of the main problems in imaging through biological tissue is autofluorescence. Coda *et al*. (*35*) showed the single-photon excitation spectrum of human colon tissue at different wavelengths. Under 435-nm single-photon excitation, colon tissue presents a very intense autofluorescence signal at the green part of the spectrum, overlapping with the light emitted from the beads or cells used in our experiment. Single-photon excitation at 435 nm is relatively similar to two-photon excitation at 800 nm, so it poses a big obstacle in the experiments because it reduces the number of photons that can reach the beads or HEK cells and it also generates undesired background light. The optical sectioning capability of TF would seem to circumvent this impediment; however, as temporally focused laser pulses propagate longer distances through scattering tissue, the resulting excitation plane becomes thicker (fig. S4) (*36*), and consequently, some autofluorescence is excited in the colon tissue, resulting in high noise levels even in the reference images taken in transmission. Despite the intense autofluorescence light emitted by the colon tissue, we succeeded in imaging both 400-nm fluorescent beads and HEK cells through 250 and 200 μm (~3 *l*_s_), respectively, obtaining high SBR values (Fig. 3). An additional image taken in a scattering phantom with fluorophores extending its entire volume confirms that TRAFIX can be used in presence of out-of-focus background fluorescence (fig. S8).

The photolocalization analysis looked at spacing between adjacent beads and cell size in a given image. It was satisfactory in the case of images through the scattering phantom obtaining deviations smaller than 3% for beads in any CR (table S3). We also obtained acceptable results in the case of cells for the image with no compression (table S2). In general, larger deviations were observed for the images through colon tissue presumably because of very low photon count reaching the detector and distortions caused by inhomogeneities in the tissue.

**Fig. 4:**
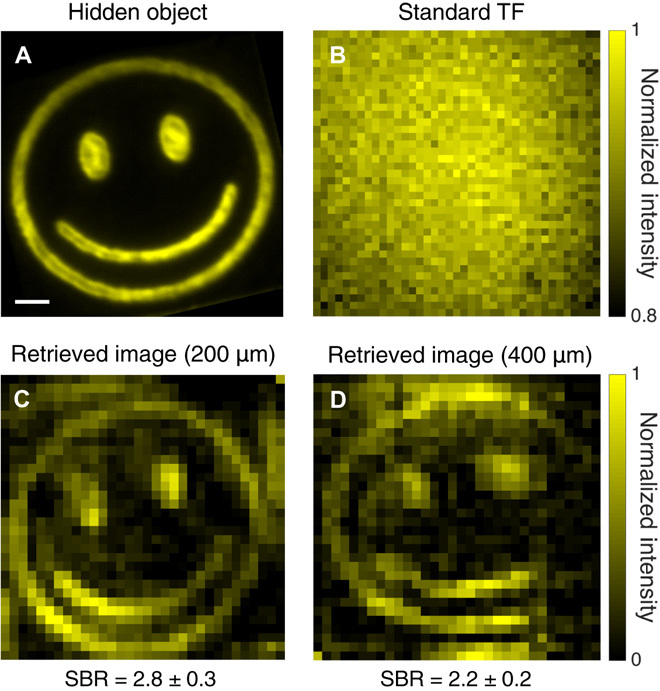
Comparison of a hidden object and the retrieved images through fixed rat brain tissue. (**A**) Reference image of a fluorescent micropattern without any scattering sample. (**B**) Image obtained by conventional TF microscopy (i.e., under uniform wide-field TF illumination with wide-field detection in epifluorescence configuration) through 400 μm of fixed rat brain tissue. (**C** and **D**) Reconstructed images obtained with TRAFIX through 200 and 400 μm of rat brain tissue, respectively. The two retrieved images were reconstructed using a full Hadamard basis containing 1024 patterns. Camera binning was set to 64 × 64, and exposure time values were (C) 0.2 s and (D) 1 s. Small intensity variations in the reconstructed images arise from inhomogeneities in the fluorescent micropattern originated in the imprinting process. Larger intensity variations are due to inhomogeneities in light transmission through the highly anisotropic scattering medium. This also applies to figs. S7, S9, S10, and S12. The SBR is shown for all reconstructed images. Scale bar, 10 μm.

We generated fluorescent micropatterns having a more detailed structure than beads or cells on a thin fluorescent film (see Materials and Methods) and imaged them through a scattering phantom (figs. S7 and S9), colon tissue (fig. S10), and fixed rat brain tissue of different thicknesses (Fig. 4). The maximum imaging depth achieved through rat brain tissue was 400 μm, which corresponds to approximately 7 *l*_s_. The improvement of TRAFIX over conventional TF microscopy becomes evident by comparing Fig. 4B with Fig. 4 (C and D).

A simple point-scanning two-photon microscope (2PM) was developed to compare the performance of TRAFIX with this widely used imaging approach (see Materials and Methods). To provide a fair comparison, we performed two experiments in which either the lateral or axial resolution of 2PM was matched to those of TRAFIX (see the Supplementary Materials). The laser power per unit area was adjusted to generate equivalent fluorescence intensity on the sample for both techniques. Exposure time and camera binning were set accordingly to perform imaging in analogous conditions. Under these conditions, images taken without scattering show equal values of SBR in the two imaging modalities (fig. S9, A and B). Figure S9 shows that SBR in 2PM degrades more rapidly than that in TRAFIX when imaging at depth. This trend was confirmed by imaging through 200 μm of unfixed human colon tissue under the previously defined imaging conditions (fig. S10). Both studies show that TRAFIX achieves between two and five times higher SBR than 2PM for the samples and depths considered in this study. Furthermore, an additional study shows that the wide-field nature of TRAFIX combined with patterned illumination results in at least three times lower photobleaching of the fluorescent sample compared to 2PM, even for laser power levels favorable to the latter (fig. S12).

Figure S13 shows how the focused spot of 2PM and various TF Hadamard patterns of TRAFIX get distorted at different depths through unfixed human colon tissue. After 400 μm, the 2PM focused spot is not discernible and turns into a complete speckle pattern. At the same depth, low-frequency Hadamard patterns retain their shape reasonably well, although having substantial intensity inhomogeneities. These results align well with the data presented by Rowlands *et al*. (*22*) using TF illumination. In these conditions, the fact that fluorescence excitation in 2PM occurs outside the expected region of focal spot suggests that TRAFIX may achieve improved imaging depths compared to 2PM, although in low resolution. It should also be noted that the axial resolution through scattering media, for the current embodiment of TRAFIX, is reduced more rapidly than that of 2PM (fig. S11). Axial confinement in TRAFIX could be improved by relying on line-scanning TF rather than wide-field TF illumination (*37*).

### Numerical simulation

**Fig. 5:**
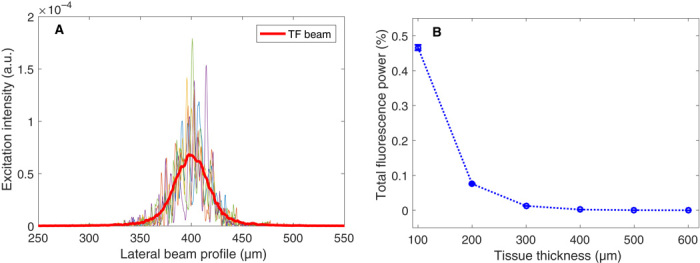
Numerical simulation of TRAFIX in scattering media. (**A**) Simulated TF laser beams at the focal plane through a 400-μm-thick brain tissue. The solid red curve indicates the smoothed-out lateral beam profile, taking all monochromatic components of the laser pulse into account. (**B**) Total fluorescence power collected with an NA = 0.8 microscope objective for different thicknesses of brain tissue. Incident laser power at sample surface is set to 100 arbitrary units (a.u.).

To illustrate how TRAFIX behaves in scattering media, we performed a simplified one-dimensional simulation of the imaging process. We took into account the propagation of TF Gaussian beams through a scattering medium and the detection of fluorescent light after backward propagation through the same medium (see the Supplementary Materials). Our simulation shows that monochromatic light propagating through a highly scattering layer, such as a 400-μm-thick brain tissue, is transformed into a spatial speckle pattern, as expected. However, one important feature of TF is that each monochromatic portion of the beam propagates through a different optical path. As identified by Papagiakoumou *et al*. (*9*), waves traveling with very different optical paths produce an overall smoothing of the beam, while slightly different optical paths produce only an anisotropic smoothing. As a result, large features of the initial beam profile are remarkably well conserved as shown in Fig. 5A. Although the main source of two-photon excitation in TRAFIX is typically generated by ballistic photons, at large depths, the excitation caused by scattered photons becomes important when compared to the highly attenuated ballistic light. As the TF beams are basically speckle free, scattering of light in this case does not heavily distort the Hadamard patterns but, in fact, contributes into making them brighter with the only disadvantage of generating softer edges in the features of the pattern.

In the second part of the simulation, we estimated the total fluorescent light that would reach our detector with respect to the total laser power deposited on the sample surface. It is clear to see in Fig. 5B that it decays markedly as the thickness of the scattering medium increases. Owing to single-pixel detection, in our experimental measurements, we could use those very few photons to form images through fixed rat brain tissue of up to 400 μm thickness.

## DISCUSSION AND CONCLUSIONS

The combination of patterned TF illumination with single-pixel detection achieves remarkable imaging depths for wide-field multiphoton microscopy as it provides a way of exciting fluorescent structures deeper inside turbid media than existing imaging techniques and can efficiently collect the emitted light in an epifluorescence configuration. We have demonstrated the effectiveness and potential of TRAFIX by imaging fluorescent beads of 400 nm in diameter and fixed HEK293T/17-GFP cells through a layer of a scattering phantom with a thickness of more than 500 μm (~4 *l*_s_), without any aberration correction, guide star, or detector placed in/behind the turbid media. We then imaged a bright custom fluorescent microstructure through rat brain tissue of hundreds of micrometers in thickness, reaching a maximum imaging depth of ~7 *l*_s_. In addition, we showed that TRAFIX works well under typical biological research conditions by imaging both fluorescent beads and HEK cells through depths of more than 3 *l*_s_ of unfixed human colon tissue and even in the presence of intense background fluorescence.

The main factor that limits imaging depth of TRAFIX is the penetration depth of the TF Hadamard patterns. Propagation of TF beams through very large distances in scattering media results in distortions on the illumination patterns mainly caused by refractive index changes in the sample. These distortions generate a basis mismatch that may result in deformities in the reconstructed images (*38*). In addition, the use of a one-dimensional diffraction grating generates horizontal shifts in the illumination Hadamard patterns that could potentially be minimized by dispersing the patterns isotropically. A future embodiment using dispersion in two dimensions with two perpendicular diffraction gratings would be an improvement. Despite these present issues, TRAFIX is capable of imaging at remarkable depths with low power per unit area over a large FOV. Its performance may be further improved by combining it with wavefront correction, making it possible to maintain spatial integrity of the illumination patterns even beyond the current limits. Furthermore, the imaging depth could be significantly improved by relying on longer wavelengths and higher-order multiphoton processes such as three-photon excitation (see the Supplementary Materials) (*22*, *39*, *40*).

The results presented here align well with previous work published in the literature such as the study by Rowlands *et al*. (*29*). They compared the penetration depth of standard TF microscopy with 2PM. A careful look at their measured modulation transfer functions (MTFs) shows a remarkably higher contrast under TF illumination at depths up to 100 to 150 μm with respect to 2PM. At larger depths, the MTF of the standard TF microscope drops markedly because of the impossibility of retaining any spatial information in the detection system. In contrast, our novel TRAFIX approach uses single-pixel detection to efficiently collect fluorescent light, extending the high performance of TF to deeper regions. Our comparison with 2PM shows that TRAFIX achieves an enhancement of up to five times in SBR when imaging through a scattering phantom and unfixed human colon tissue. Moreover, photobleaching of the sample is substantially reduced as a result of using wide-field TF patterned illumination rather than a focused high-intensity beam (see the Supplementary Materials). Image resolution and SBR can be further improved by using new approaches such as digital microscanning (*41*).

Here, we also demonstrate that compressive sensing measurements are possible in TRAFIX by showing reconstructed images a posteriori. However, in an actual compressive sensing measurement with no a priori knowledge of the sample, choosing the most appropriate illumination patterns is critical. Since the amount of information carried by each pattern is uneven, it is important to wisely choose the order in which they are projected to optimize image quality and acquisition speed (*42*).

In the present embodiment of TRAFIX, the acquisition speed is mainly limited by the exposure time of the EMCCD camera and the slow refresh rate of the SLM (see the Supplementary Materials). A typical image obtained using a full basis of 1024 patterns (32 pixels by 32 pixels) through a moderate thickness of a scattering sample currently takes ~5 min. This time is increased to up to 1 hour when imaging in high resolution (64 pixels by 64 pixels) with a full basis through the most challenging conditions shown in this article. To speed imaging up, the EMCCD camera would be replaced with a fast, sensitive photodetector such as a photomultiplier tube (PMT) and the imaging speed would no longer be limited by the exposure time of the detector. In addition, as TRAFIX currently uses binary Hadamard patterns, the SLM may be replaced with a significantly faster digital micromirror device, which may run at tens of kilohertz. These changes, combined with the new advances in compressive imaging, suggests that frame rates for 128-pixel by 128-pixel images can be increased to more than ~30 Hz (*43*, *44*), enabling studies in time-varying turbulence (*45*).

Since our scheme can be easily implemented in a standard multiphoton microscope, we believe that one of its main applications will be in the field of optogenetics where it would lend itself to achieve long-term simultaneous imaging and photoactivation of neuronal networks with minimal photodamage deep inside the brain. Last, as the polarization state of the illumination light does not change over propagation in scattering media through the range of distances normally considered for imaging (see the Supplementary Materials) (*46*), TRAFIX could also be combined with polarization-resolved imaging techniques (*47*).

In summary, TRAFIX is a novel approach for deep multiphoton imaging that presents an increased SBR compared to the ubiquitous 2PM while also reducing photobleaching of the sample. In addition, the almost speckle-free propagation of TF illumination patterns suggests that TRAFIX may surpass the maximum imaging depth limit of 2PM and may be very beneficial for long-term biological studies, particularly in neuroscience.

## MATERIALS AND METHODS

### Experimental setups

#### TempoRAl Focusing microscopy with single-pIXel detection

An illumination laser (Coherent Chameleon Ultra II) delivers 140-fs pulses with an 80-MHz repetition rate, up to 4-W average output power at a variable central wavelength between 680 and 1080 nm. The central wavelength of the laser was set to 800 nm for all the experiments performed. The illumination beam was expanded four times to cover the active area of a phase-only SLM (LCOS-SLM, Hamamatsu Photonics). The SLM was then imaged onto a blazed reflective grating (1200 g/mm) with a 4*f* (*f* = 400 mm) telescope to create wide-field TF illumination. The first diffraction order from the SLM was transmitted through an iris in the telescope, while all other orders were blocked. The beam was diffracted from the grating, and all wavelengths were collimated with an *f* = 400 mm lens relayed onto the back focal aperture of the illumination objective. Two different illumination objectives were used in this work. A water dipping objective (40× NA = 0.8; Nikon), which is enclosed in a custom-made chamber filled with water, generates a TF illumination plane with a size of 90 μm by 90 μm. The highest average laser power per unit area used in this configuration is 64 ± 5 μW/μm^2^. An air objective (20× NA = 0.75; Nikon) was used for additional studies presented in the Supplementary Materials, as accordingly specified. Backscattered fluorescent light propagated through the turbid media and was collected by an EMCCD camera run without amplification (iXon^EM^+ 885, Andor Technology) via the same illumination objective in epifluorescence configuration. To provide reference images for this paper, forwardly emitted photons from the sample were collected by a CCD camera (Clara, Andor Technology) in transmission via a long working distance air objective (100× NA = 0.7; Mitutoyo). Appropriate short-pass filters were used to reject the illumination laser at 800 nm and transmit fluorescence below 700 nm. In contrast to other single-pixel imaging approaches (*25*, *26*), the EMCCD camera with 64 × 64 binning was used as a bucket detector instead of using a single-element detector such as a PMT or an avalanche photodiode. Using high binning helps reducing the effect of readout noise. All objectives, samples, and cameras were attached on the body of an inverted microscope (Eclipse Ti, Nikon) accordingly.

#### Point-scanning two-photon microscope

The 2PM shares the same setup as TRAFIX, except for the diffraction grating and a lens that are replaced with a mirror to obtain a focused beam on the focal plane of the illumination objective. A Nikon 20× NA = 0.75 air objective was used for all experiments. A variable iris was used to adjust the size of the focused spot. An X-Y-Z motorized stage (Nano-LP200, Mad City Labs) scanned the sample in a stepwise motion across the fixed focused beam covering the entire FOV. The same binned EMCCD camera run with no amplification (iXon^EM^+ 885, Andor Technology) collected the fluorescent light emitted by the sample.

### Fluorescent and scattering samples

#### Fluorescent layer and fluorescent micropattern

A 200-nm-thin layer of super-yellow polymer spin-coated on a glass coverslip was used to characterize the profile and depth resolution of the TF beam (see the Supplementary Materials). It was also used to generate a fluorescent micropattern that was then imaged through scattering media. The negative of a pattern of interest was encoded on the SLM, and the thin fluorescent layer was placed at the focal plane of the microscope without any scattering layer. The laser power was set to the maximum, and the negative pattern was photobleached on the fluorescent film. Therefore, the only portion of the FOV that remained fluorescent was exactly the desired micropattern.

#### Fluorescent beads

Green fluorescent polymer microspheres (G400, Duke Scientific) with a diameter of 0.39 μm were used to test the performance of the imaging system. A very small amount of beads was deposited on a glass coverslip and placed on top of the scattering samples to image them through the turbid media.

#### HEK cells

HEK293T/17-GFP cells were used to demonstrate the capability of the microscope in imaging real biological samples through scattering. HEK293T/17 cell line obtained from American Type Culture Collection was cultured in Dulbecco’s modified Eagle’s medium GlutaMAX-I supplemented with 10% fetal bovine serum and 1% penicillin/streptomycin and was transfected using TransIT-LT1 transfection reagent with the Vesicular Stomatitis Virus glycoprotein (VSV-G) pseudotyped lentivirus vector and the packaging plasmid psPAX2 to deliver the plasmid pLenti-GFP-Puro. Regarding the sample preparation, the HEK293T/17-GFP cells were replated on World Precision Instruments FluoroDish poly-d-Lysine–coated cell culture dishes at a low density to achieve ideal imaging conditions. The day after plating, before imaging, the HEK293T/17-GFP cells were fixed in a phosphate-buffered saline 4% paraformaldehyde solution. The scattering samples were attached directly on the bottom of the dishes containing the cells.

#### Scattering phantom

Polystyrene beads of 1 μm in diameter were used as scatterers to simulate a turbid sample. The beads were purchased in a 1% concentration solution in water (Microbead NIST Traceable Particle Size Standard, 1.00 μm; Polysciences). The solution was thoroughly stirred in a vortex mixer and then mixed with a 1% solution of agarose in water (preheated above melting point). Agarose and beads were mixed in the vortex mixer again and placed into sample wells of variable height. The wells consisted of a 100-μm glass slide with multiple 90-μm vinyl spacers stacked on top of each other. An additional coverslip was placed on top to seal the well. The concentration of polystyrene beads in the sample was chosen to roughly match the scattering coefficient of real biological tissue (*48*). Using an on-line Mie scattering calculator (*49*), we determined the reduced scattering coefficient of our phantom to approximately be $\mu_{s}^{\prime }\approx 7.5\;{\text{cm}}^{-1}$, corresponding to a mean free path of about $l_{s}=(1-g)/\mu_{s}^{\prime }\approx 140\;\mu m$, where *g* is the anisotropy factor (*g* ~ 0.9 for most biological tissue at the wavelength considered in this investigation). Different scattering phantoms were used in some experiments in the Supplementary Materials as appropriately specified.

#### Rat brain tissue

Similar to our previous work (*50*), rat brain tissue was obtained from adult Sprague Dawley rats, in accordance with the UK Animals (Scientific Procedures) Act 1986. It was fixed and then sectioned into slices at thicknesses of 200 and 400 μm. The mean free path of the rat brain tissue was estimated by measuring the ratio of the incident laser intensity *I*_0_ with the intensity of the ballistic photons *I*_B_ and by applying an exponential law *I*_B_ = *I*_0_ exp(− *L*/*l*_s_), where *L* is the thickness of the brain tissue (*18*). The obtained value of *l*_s_ = 55 ± 9 μm is consistent with other measurements reported in the literature for rat (*51*) and mouse brains (*52*).

#### Colon tissue

Thin fragments of unfixed normal human colon tissue were used as scattering samples. They were stored in a freezer at −80°C and mounted between two coverslips. Their thickness ranged from 200 to 250 μm, and their reduced scattering coefficient was approximately 12 cm^−1^ (*53*, *54*), giving a mean free path of *l*_*s*_ ≈ 85 μm. The colon tissue sample used in this study was obtained from the Tayside Tissue Bank, Ninewells Hospital and Medical School, Dundee (tissue request no. TR000289) with appropriate ethical permission.

### Image quality quantification

#### Signal-to-background ratio

SBR is defined here as SBR = μ_sig_/μ_bg_, where μ_sig_ and μ_bg_ are the average values of the signal and the background, respectively. In the case of cells and fluorescent micropatterns, two small regions of interest were defined—one containing fluorescent structures and the other corresponding to the background. In the images of beads, the highest-intensity pixels in the beads were used as signal value, and the maximum intensity pixels in the rest of the image were used as background noise. Uncertainty is given by the SD of all averaged measurements. All values of SBR presented in this work are summarized in table S1.

#### Cell size

As the cells used here appear approximately spherical, their diameter was estimated by taking a measurement of their size in two orthogonal directions and averaging the obtained values. Their diameter was determined by fitting a Gaussian function to the intensity values and measuring its full width at half maximum. Table S2 shows the diameters of the cells that appear in the different images presented in this work. Deviations in cell size between reconstructed and reference images are expressed as a percentage error.

#### Bead spacing

The central pixel for each individual bead was located, and the distance between beads was measured in the reference image and in all retrieved images with different CRs (table S3). Deviations in bead spacing between reconstructed and reference images are expressed as a percentage error.

## Supplementary Material

http://advances.sciencemag.org/cgi/content/full/4/10/eaau1338/DC1

## SUPPLEMENTARY MATERIALS

Supplementary material for this article is available at http://advances.sciencemag.org/cgi/content/full/4/10/eaau1338/DC1 Note S1. Numerical simulations Note S2. Single-pixel detection and compressive sensing Note S3. Microscope characterization Note S4. Comparison between TRAFIX and point-scanning two-photon microscopy (2PM) Note S5. Polarization state evaluation Fig. S1. Numerically simulated TF laser beam propagating through 400 μm of brain tissue. Fig. S2. Properties of a numerically simulated TF laser beam through brain tissue. Fig. S3. Effect of scattering on the beam profile with and without TF. Fig. S4. Depth profile of a TF beam through a scattering phantom. Fig. S5. Characterization of a TF beam through a scattering phantom. Fig. S6. Images of fluorescent microscopic samples without scattering. Fig. S7. Comparison of a hidden object and retrieved images through a scattering phantom with different resolution. Fig. S8. Image of 4.8-μm fluorescent beads in a volumetric scattering phantom. Fig. S9. Comparison of SBR of TRAFIX and point-scanning two-photon microscopy (2PM) at depth. Fig. S10. Comparison of TRAFIX and point-scanning two-photon microscopy (2PM) through human colon tissue. Fig. S11. Axial confinement in TRAFIX and point-scanning two-photon microscopy (2PM). Fig. S12. Photobleaching comparison of TRAFIX and point-scanning two-photon microscopy (2PM). Fig. S13. Effect of scattering on illumination beams in point-scanning two-photon microscopy (2PM) and TRAFIX. Fig. S14. Effect of turbid media on light polarization. Table S1. SBR measured for all the images shown in this work. Table S2. Cell diameters of all images shown in this work. Table S3. Beads spacing corresponding to all images shown in this work. References (*55*–*61*)
