# Supplementary material for: Wide-field multiphoton imaging through scattering media without correction
Source: Sci Adv. 2018 Oct 12;4(10):eaau1338. doi: 10.1126/sciadv.aau1338 (PMC6184782; doi:10.1126/sciadv.aau1338)
Supplement: http://advances.sciencemag.org/cgi/content/full/4/10/eaau1338/DC1 [file supp_4_10_eaau1338__index.html]

Science Advances | Science Advances

## Supplementary Materials

**This PDF file includes:**

- Note S1. Numerical simulations
- Note S2. Single-pixel detection and compressive sensing
- Note S3. Microscope characterization
- Note S4. Comparison between TRAFIX and point-scanning two-photon microscopy (2PM)
- Note S5. Polarization state evaluation
- Fig. S1. Numerically simulated TF laser beam propagating through 400 μm of brain tissue.
- Fig. S2. Properties of a numerically simulated TF laser beam through brain tissue.
- Fig. S3. Effect of scattering on the beam profile with and without TF.
- Fig. S4. Depth profile of a TF beam through a scattering phantom.
- Fig. S5. Characterization of a TF beam through a scattering phantom.
- Fig. S6. Images of fluorescent microscopic samples without scattering.
- Fig. S7. Comparison of a hidden object and retrieved images through a scattering phantom with different resolution.
- Fig. S8. Image of 4.8-μm fluorescent beads in a volumetric scattering phantom.
- Fig. S9. Comparison of SBR of TRAFIX and point-scanning two-photon microscopy (2PM) at depth.
- Fig. S10. Comparison of TRAFIX and point-scanning two-photon microscopy (2PM) through human colon tissue.
- Fig. S11. Axial confinement in TRAFIX and point-scanning two-photon microscopy (2PM).
- Fig. S12. Photobleaching comparison of TRAFIX and point-scanning two-photon microscopy (2PM).
- Fig. S13. Effect of scattering on illumination beams in point-scanning two-photon microscopy (2PM) and TRAFIX.
- Fig. S14. Effect of turbid media on light polarization.
- Table S1. SBR measured for all the images shown in this work.
- Table S2. Cell diameters of all images shown in this work.
- Table S3. Beads spacing corresponding to all images shown in this work.
- References (*55*–*61*)

Download PDF

**Files in this Data Supplement:**

- Adobe PDF - aau1338\_SM.pdf
