# Supplementary material for: Wide-field multiphoton imaging through scattering media without correction
Source: Sci Adv. 2018 Oct 12;4(10):eaau1338. doi: 10.1126/sciadv.aau1338 (PMC6184782; doi:10.1126/sciadv.aau1338)
Supplement: http://advances.sciencemag.org/cgi/content/full/4/10/eaau1338/DC1 [file aau1338_SM.pdf]

Science Advances
AAAS

advances.sciencemag.org/cgi/content/full/4/10/eaau1338/DC1

Supplementary Materials for

Wide-field multiphoton imaging through scattering media without correction

Adrià Escobet-Montalbán, Roman Spesyvtsev, Mingzhou Chen, Wardiya Afshar Saber, Melissa Andrews, C. Simon Herrington, Michael Mazilu, Kishan Dholakia*

*Corresponding author. Email: kd1@st-andrews.ac.uk

Published 12 October 2018, *Sci. Adv.* **4**, eaau1338 (2018)
DOI: 10.1126/sciadv.aau1338

This PDF file includes:

Note S1. Numerical simulations
Note S2. Single-pixel detection and compressive sensing
Note S3. Microscope characterization
Note S4. Comparison between TRAFIX and point-scanning two-photon microscopy (2PM)
Note S5. Polarization state evaluation
Fig. S1. Numerically simulated TF laser beam propagating through 400 μm of brain tissue.
Fig. S2. Properties of a numerically simulated TF laser beam through brain tissue.
Fig. S3. Effect of scattering on the beam profile with and without TF.
Fig. S4. Depth profile of a TF beam through a scattering phantom.
Fig. S5. Characterization of a TF beam through a scattering phantom.
Fig. S6. Images of fluorescent microscopic samples without scattering.
Fig. S7. Comparison of a hidden object and retrieved images through a scattering phantom with different resolution.
Fig. S8. Image of 4.8-μm fluorescent beads in a volumetric scattering phantom.
Fig. S9. Comparison of SBR of TRAFIX and point-scanning two-photon microscopy (2PM) at depth.
Fig. S10. Comparison of TRAFIX and point-scanning two-photon microscopy (2PM) through human colon tissue.
Fig. S11. Axial confinement in TRAFIX and point-scanning two-photon microscopy (2PM).
Fig. S12. Photobleaching comparison of TRAFIX and point-scanning two-photon microscopy (2PM).
Fig. S13. Effect of scattering on illumination beams in point-scanning two-photon microscopy (2PM) and TRAFIX.
Fig. S14. Effect of turbid media on light polarization.
Table S1. SBR measured for all the images shown in this work.
Table S2. Cell diameters of all images shown in this work.
Table S3. Beads spacing corresponding to all images shown in this work.
References (*55*–*61*)

Note S1. Numerical simulations

Two-photon temporal focusing microscopy

We numerically simulate a temporal focusing (TF) Gaussian beam propagating through turbid media and exciting a uniform fluorescent layer. Laser pulses centered at 800 nm ($\Delta\lambda = \pm 3.5$ nm) are used as an excitation source. A layer of turbid tissue is simulated by mixing an aqueous medium with randomly distributed dielectric spheres of 2 μm diameter at a concentration of 1 sphere per 1000 μm^3. The refractive index of the scattering spheres is 0.1 higher than the surrounding media. Numerical simulations are carried out only in one dimension to reduce the required computation power. Similarly to the grating in the experiment, the spectral components in the laser pulse are dispersed and expanded to fill the back aperture of a microscope objective (NA = 0.8) as shown in fig. S1 (a). Assuming that the laser pulse contains 70 spectral components at different wavelengths with a step of 0.1 nm, each monochromatic component of the pulse propagates through a different region of scattering tissue and arrives at the same position on the focal plane using the split step approach implemented in Matlab (2016a) (*55, 56*). Back scattering is neglected here while the attenuation coefficient of turbid media is set to 4.45×10^3 m^{-1}, experimentally estimated by measuring the laser power before and after tissue samples (*57*). At the focal plane, each spectral component forms a speckle pattern as shown in fig. S1 (b). These speckle patterns are significantly spatially correlated but shifted from each other since each different wavelength travels along a slightly different optical path through the turbid medium. As expected, temporally focusing these speckle patterns results in a distinct 'smooth' excitation beam at the focal plane as shown by the solid red curve in fig. S1 (b). According to the numerical simulation results, the total power delivered through the tissue decreases exponentially with the thickness of tissue as shown in fig. S2 (a). Figure S2 (b) also shows that the beam's full width at half maximum (FWHM) at the TF plane increases slightly when the beam is focused through a thick layer which would

result in a slight distortion of the Hadamard patterns projected in the experimental measurements. The excited fluorescent photons have to propagate through the turbid tissue before they can be collected by the same microscope objective and therefore the total fluorescence signal becomes very weak for a thick tissue layer compared to the initial illumination power as shown in fig. S2 (c).

Three-photon temporal focusing microscopy

We also numerically simulated three-photon TF microscopy using laser pulses centered at 1700 nm ($\Delta\lambda = \pm 3.5$ nm) to excite a fluorescent layer under the same slice of tissue. As the attenuation length at 1700 nm is almost doubled for the same brain tissue (*39*), the attenuation coefficient is now set to 2.5×10^3 m^{-1}. Assuming all other parameters are the same, three-photon TF beams show a better penetration through the layer of tissue. Figure S1 (d) shows that the overall smoothed out speckle pattern reaching the focal plane resembles the initial Gaussian beams more than the one shown in (b). Additionally, thanks to using longer wavelengths, the amount of TF laser power reaching focus is considerably higher than in the previous case fig. S2 (a). As the FWHM of the overall smoothed out beam is thinner (fig. S2 (b)), the shape of the illumination Hadamard patterns would be distorted by a smaller amount than in two-photon excitation. This, combined with the fact that its intensity is higher (fig. S1 (d)), would make three-photon TRAFIX capable of penetrating much deeper in biological tissue. For the same fluorescence signal level detection threshold, TRAFIX in three-photon mode can penetrate nearly 1.5 times deeper into the tissue than in two-photon mode.

Note S2. Single-pixel detection and compressive sensing

Compressive sensing is a signal processing technique for acquiring and reconstructing signals with fewer measurements than required by the Nyquist sampling theorem. The descrip-

tion of single-pixel detection and compressive sensing presented in this section is based on the work done by Tajahuerce *et al.*, Durán *et al.*, Candès *et al.*, Tropp *et al.*, and Olivas *et al.* (*25, 26, 30, 31, 58*).

Any signal or image, x, can be expanded as a sum of weighted basis functions

$$x = \sum_{n=1}^{N} \omega(n)\psi_n \tag{S1}$$

where ψ_n are the basis functions, $\omega(n)$ are the weighting coefficients and *N* is the total number of pixels. In order to represent an image with perfect accuracy, the number of basis functions used in the reconstruction should be the same as the total number of pixels in the image. It is also well known that the Nyquist sampling theorem states that the sampling rate must be at least twice the highest frequency that needs to be resolved. However, if the image is sparse, compressive sensing can break this limit and reconstruct images or signals with far fewer samples or measurements than required by the Nyquist theory. Sparse images can be defined as images in which most weighting coefficients are small and only a few of them are relatively large so image quality is not lost by neglecting the smallest ones. Therefore, there is no need to measure all the basis coefficients, instead, one can make only a few random measurements that have equal probability of obtaining relevant data and with the information captured, the image can be reconstructed. In terms of Eq. S1, a good image can be reconstructed by only summing *K* weighted basis functions with *K* much smaller than *N*

$$x \cong \sum_{l=1}^{K} \omega(l)\psi_l \tag{S2}$$

The reconstruction process can be further optimized by using algorithms as described below.

Single-pixel detection and compressive sensing in TRAFIX

In TRAFIX, the sample is illuminated by microstructured light patterns, each corresponding to a basis function, and the total intensity emitted by the sample for each pattern is measured with a photodetector. Different sets of bases can be used to perform a single-pixel measurement. Olivas *et al.* compared the performance of a digital (Hadamard), a grayscale (discrete cosine transform) and a random (Noiselet) set of bases. According to their work, a random set of bases like the Noiselet, provides a more uniform sampling and higher spatial frequency information is present in the reconstructed images. However, more images are needed to converge to a visually appealing solution. They also stated that spatially structured patterns such as the Hadamard or discrete cosine transform, interact with the structure of the sample and certain weighting coefficients stand out, which in most occasions, helps reaching more easily a solution that looks nicer. In their study, they concluded that in general, the Hadamard basis provides better results (*58*).

In our TRAFIX microscope, we performed single-pixel measurements with an *a posteriori* approach to compressive sensing. We considered a basis formed of Hadamard patterns whose entries are either 1 or -1. Two important properties of Hadamard matrices are that their rows are orthogonal and they fulfill the following condition

$$HH^T = nI_n \tag{S3}$$

where *H* is a Hadamard matrix and *I* an identity matrix of dimension n. Hadamard matrices were encoded on a SLM and the corresponding patterns were projected onto the sample after propagating through turbid samples. As the number of pixels in the reconstructed image is determined by the size of the basis, two different sets of Hadamard bases containing 1024 and 4096 elements were used to obtain images of 32 pixels by 32 pixels and 64 pixels

by 64 pixels, respectively. Since Hadamard matrices are made of -1 and 1, each pattern had to be split into a sequence of two complementary patterns, one containing the positive portion and the other one the negative portion of the pattern. Then, measurements taken with the negative part of the basis were subtracted from the positive portion obtaining a weight coefficient for each element of the Hadamard basis. The background was subtracted from each measurement. Once all patterns had been projected and their weights had been determined, the final image could be reconstructed by summing them up as described in Eq. S1. However, if only a few patterns were used in the reconstruction, we could obtain different compression ratios (CRs) (*26*). Reconstruction with fewer patterns is *equivalent* to taking very few measurements in the first place. In order to optimize the reconstruction process we implemented an orthogonal matching pursuit (OMP) algorithm in the same way as Tropp *et al.* and Phillips (*30, 59*).

Note S3. Microscope characterization

Beam profile

Before taking any images through scattering media, the performance of the microscope was characterized. Firstly, the profile of a TF beam was imaged on the reference arm using the fluorescent polymer layer with no scattering (fig. S3 (a)) and with a 900 µm thick scattering phantom ($l_s \approx 250$ µm) (fig. S3 (b)). The profile after scattering appears dimmer but its shape remains practically unchanged. Then, the diffraction grating was replaced with a mirror in order to obtain a wide-field image without TF. In this case the beam profile after scattering turns into a speckle pattern (fig. S3 (d)). These results are in accordance with the ones presented in (*9*) and show the resilience of TF to speckle formation after propagation through scattering media.

Axial resolution

The axial resolution of the microscope was characterized by moving the sample axially across the focal plane with a stepper motor (Newport Universal Motion Controller/Driver, ESP300) and measuring the total intensity coming from the thin fluorescent layer at different axial positions separated 250 nm from each other. A water dipping Nikon 40× NA = 0.8 was used for this investigation. Without scattering, the thinnest excitation plane achieved was measured to be 4.7 ± 0.5 μm. The same measurement was performed through various thicknesses of the scattering phantom, used in the previous section, to determine how the depth resolution changes as we image deeper into turbid samples (fig. S4).

Figure S5 (a) shows that the depth resolution of TRAFIX deteriorates as the beam propagates deeper inside a turbid medium but it only decreases by a factor of 2.5 after propagating through more than 700 μm of the medium. In addition, the intensity of the light emitted by the excited plane decreases exponentially (fig. S5 (b)). The results presented in this section are in accordance with our simulations (Fig. 5) and the data presented by studies of Dana *et al.* (*36*) and Papagiakoumou *et al.* (*60*).

Imaging without scattering

Prior to imaging through turbid samples, the performance of the microscope was tested without scattering. Figure S6 shows the images of the fluorescent beads and HEK cells obtained with different CR. Image quality in the case of beads remains practically the same, both visually and in terms of signal-to-background ratio (SBR), for all levels of compression. High CR could not be achieved for cell imaging mainly because the images are not sparse enough.

Lateral resolution through scattering

According to Nyquist's sampling criterion applied to imaging, in a standard microscope a small detail of a specimen has to be sampled by at least two pixels on a camera in order to obtain a well-resolved image. Therefore, we define the lateral resolution in TRAFIX as the size of two pixels in the reconstructed images.

A fluorescent micropattern was imaged through 490 μm of scattering phantom with two different sets of Hadamard bases to show the increase in resolution when using a larger number of patterns (fig. S7). With a FOV of approximately 90 μm by 90 μm and using a 32-pixel by 32-pixel basis (1024 patterns), the lateral resolution is 5.6 μm; and using a 64-pixel by 64-pixel basis (4096 patterns) it becomes 2.8 μm. The resolution can also be improved by making the FOV smaller using higher magnification optics.

Imaging volumetric fluorescent samples

A scattering phantom with fluorophores extending its entire volume was imaged to determine the performance of TRAFIX in imaging in presence of intense out-of-focus fluorescent light. The phantom contained 0.4 μm and 4.8 μm diameter fluorescent beads, and 1 μm diameter polystyrene beads embedded in 1.5 % agarose. The 0.4 μm fluorescent microparticles contribute to creating a fluorescent background and the 4.8 μm beads simulate features of interest. This experiment was done with a Nikon 20× NA = 0.75 air objective.

Figure S8 shows an image of the phantom at a depth of ∼300 μm. We remark that no reference images are shown because this is a three-dimensional sample and the imaged plane is not accessible from the reference imaging system. As the axial confinement of the TF illumination plane with the present objective at a depth of ∼300 μm is ∼20 μm (See section S4), 4.8 μm beads out of the focal plane generate a strong fluorescent background. However, beads located at the focal plane can still be clearly distinguished by simply adjusting the gray levels of the image.

Axial resolution can be enhanced by changing imaging parameters such as magnification, NA or pulse duration (36), or by relaying on line-scanning TF illumination instead of wide-field.

Acquisition time

The total acquisition time of TRAFIX can be estimated with the equation $T = 2n^2(t_{exp} + t_{SLM})$ given in the manuscript. For example, a typical image through a scattering medium such as fig. S10 (d), was obtained using a full basis of 1024 patterns (32 pixels by 32 pixels) with an exposure time of 0.1 s per pattern and 0.03 s refresh time of the spatial light modulator (SLM). Therefore, the total acquisition time was approximately $T = 2 \cdot 1024 \cdot (0.1 + 0.03) = 266s \approx 5\,\text{min}$. This time is increased when imaging deeper in biological tissue or using dimmer fluorescent samples because the exposure time of the camera has to be increased. Increasing the total number of patterns to achieve higher resolution also increases acquisition time. The longest duration experiment (Fig. 3 (d)) took approximately 1 hour because of the high resolution (64 pixels by 64 pixels) and of the low cellular fluorescence (HEK cells).

In this present embodiment of TRAFIX, the acquisition speed is mainly limited by the low sensitivity and readout speed of the EMCCD camera and the slow refresh rate of the SLM (~30 Hz real-time rate). To speed up the imaging, the EMCCD camera would be replaced with a fast, sensitive photodetector such as a photomultiplier tube (PMT), often used for fast imaging in standard point-scanning two-photon microscopy (2PM). 2PM using a PMT routinely achieves frame rates of 15 Hz for images containing 1M pixels (e.g. used on a Nikon A1R HD microscope). The pixel dwell time is thus approximately 64 ns/pixel. In such a case, the imaging speed would no longer be limited by the exposure time of the detector. The next issue for improving acquisition time is the SLM. As TRAFIX currently uses binary Hadamard patterns, the SLM may be replaced with a significantly faster digital micromirror device, which may run at tens of kilohertz. In this way, the acquisition speed could be dramatically increased

to real-time frame rate imaging. Using the noted equation, it can be shown that a state-of-the-art DMD could achieve frame rates of ∼10 Hz for a resolution of 32 pixels by 32 pixels, ∼3 Hz for 64 pixels by 64 pixels and ∼0.7 Hz for 128 pixels by 128 pixels assuming measurements with full bases. Imaging speed can be further increased by relying on compressive sensing. The effectiveness of compressive sensing is determined by the sparsity in the datasets, a high resolution image can in general be compressed to a higher extent than a low resolution one (*42*). Therefore, increasing the size of the pattern basis makes it possible to achieve a higher compression. Compression of 1% and even higher has consistently been reported in the literature (*58*) suggesting that frame rates for 128-pixel by 128-pixel images can easily be increased to over ∼30 Hz (*43, 44*), enabling studies in time varying turbulence (*45*).

Image reconstruction in the current study is performed offline on a computer with an Intel Core i7-6700K 4GHz processor and a RAM of 32 GB. The computer processing time depends on the level of sophistication of the algorithm. For example, an image can be reconstructed in 5 s by simply using eq. S1. If noise reduction or compressed reconstruction is needed, longer processing times are typically required. The image reconstruction time using an orthogonal matching pursuit algorithm takes about 30 s for 32-pixel by 32-pixel images and it scales with the number of patterns used in the image reconstruction. This can be reduced by use of new algorithms to achieve fast real-time reconstruction at video rate (*43*).

Note S4. Comparison between TRAFIX and point-scanning two-photon microscopy (2PM)

Signal-to-background ratio (SBR) at depth

To compare the performance of TRAFIX and 2PM in imaging at depth, two experiments were carried out. They consisted in imaging fluorescent micropatterns at different depths through a scattering phantom ($l_s \approx 100\,\mu\text{m}$) and comparing the signal-to-background ratio

(SBR) of the acquired images.

An underfilled Nikon 20× NA = 0.75 air objective was used for the studies below to equate lateral and axial resolution for both approaches.

- Experiment 1: comparable lateral resolution. Firstly, the back aperture of the objective was filled so as to produce a focused spot of 3.75 μm diameter to make it similar to the lateral resolution of TRAFIX (FOV of 84 μm by 84 μm using an illumination basis size of 1024). We term this 2PM-Lateral.

- Experiment 2: comparable axial resolution. Here, the NA of the illumination beam was increased to improve the axial resolution of 2PM to 8.4 μm, which was the same as for TRAFIX (fig. S11 (c)). We term this 2-PM-Axial.

The laser power was set to the maximum for TRAFIX, obtaining a power per unit area and pattern on the sample of 0.068 mW/ μm^2·pattern. The total accumulated power per unit area after a measurement with the full basis was 69 mW/ μm^2. The laser power per unit area was then adjusted to generate approximately the same fluorescence intensity in 2PM as the total fluorescence intensity generated by TRAFIX after 1024 patterned illuminations. This was 2.2 mW/ μm^2. The exposure time of the camera was set to the same value for each pixel in 2PM scan as for each TRAFIX pattern to make the experiment fully comparable. As expected, in the experiments without scattering, the SBR was equivalent for both TRAFIX and 2PM (fig. S9). All images were obtained with different micropatterns (smiley faces) to avoid the effect of photobleaching in the SBR calculation.

The results for both experiments are shown together in fig. S9. We confirm that the SBR in 2PM degrades more rapidly than for TRAFIX when imaging at depth. Furthermore, we also performed these two experiments in exactly the same conditions through 200 μm of unfixed human colon tissue. Figure S10 also shows a higher SBR for TRAFIX at depth. These results show that TRAFIX achieves between 2 and 5 times higher SBR than 2PM for the samples

and depths considered in this study. As described in the article, this is due to the resilience of TF beams to speckle formation and the fact that the temporal profile of femtosecond pulses is distorted to a lower extent compared to the spatial profile.

Axial confinement

The axial confinement of TRAFIX and 2PM was compared through different layers of a scattering phantom having a scattering mean free path of $l_s \approx 150\ \mu\mathrm{m}$ and using a Nikon 20× NA = 0.75 objective.

Starting from the same axial confinement without scattering, fig. S11 shows that TRAFIX (or equivalently, wide-field TF) degrades faster than 2PM. The axial extent of the illuminated plane in TRAFIX increases $\sim$2.6 times after $\sim$2.4 scattering mean-free-path lengths while in 2PM it only increases by $\sim$1.8 times. This behaviour for TF illumination has already been identified by Dana and Shoham (36). It is also worth remarking that 2PM has better axial confinement than TRAFIX because the latter is currently based on wide-field TF illumination. To achieve the same axial confinement as 2PM, fast line-scanning TF illumination should be used to create the illumination patterns in TRAFIX (*37*).

Photobleaching

When imaging biological samples, photobleaching and photodamage caused by the high irradiation intensities of the illuminating light need to be carefully considered. The wide-field TF nature of TRAFIX avoids the need of spatially focusing a laser beam on the sample resulting in a reduced illumination power per unit area.

The use of patterned illumination is also beneficial for imaging sensitive biological samples since the total illumination energy is distributed over many low intensity exposures. Even though the total accumulated power per unit area in TRAFIX may be higher than in 2PM, it has

been shown that by delivering the same total light dosage in a sequential rather than instantaneous manner, photobleaching and photodamage are drastically reduced (*61*).

To verify this, we performed an experiment in which we sequentially imaged two fluorescent micropatterns without scattering with TRAFIX and with 2PM. The illumination power per unit area of TRAFIX was set to 0.034 mW/ μm^2·pattern and the resulting accumulated power per unit area after projecting 1024 patterns was 35 mW/ μm^2. We determined that in order to have the same total fluorescence count (i.e. equivalent image quality) the laser power per unit area of 2PM in this case should be 1.1 mW/ μm^2. We observed that under this power per unit area, the fluorescent micropattern was severely damaged so we decided to perform this experiment with half of that power, i.e. 0.55 mW/ μm^2.

Figure S12 (a) and (b) show six images acquired sequentially with TRAFIX and 2PM, respectively. It is clear to see that the image quality of 2PM degrades much faster than that of TRAFIX even under lower laser power per unit area than required to obtain the same SBR. Figure S12 (c) shows the fluorescence intensity decay in both techniques. Under more favorable conditions compared to TRAFIX, 2PM photobleaching is still over three times greater. Thus, this highlights a further major advantage of the TRAFIX approach.

Effect of scattering on excitation beams

A further experiment was performed to determine the effect of scattering on the illumination patterns of TRAFIX and 2PM.

A fluorescent layer placed on top of the colon tissue was illuminated with various TRAFIX patterns and the 2PM focused beam, and imaged from the reference arm. Figure S13 shows the illumination patterns without scattering and through 200 μm and 400 μm of colon tissue. It can be seen that the 2PM focused spot turns into a speckle pattern after $\sim$400 μm while low frequency Hadamard patterns remain relatively well conserved though with important brightness inhomogeneities.

Note S5. Polarization state evaluation

A scattering phantom and rat brain tissue were illuminated with linearly polarized light, and the polarization state of the transmitted light was analyzed using a linear polarizer and a power meter. The degree of linear polarization [$DOLP = (I_{\parallel} - I_{\perp})/(I_{\parallel} + I_{\perp})$] was measured in all three cases obtaining 0.999 for the incident light, and 0.978 and 0.964 for the transmitted light through the scattering phantom and rat brain tissue, respectively. Figure S14 shows that the measured data clearly obeys Malus' Law. In the figure, intensity was normalised taking into account the transmittance of the different samples and the polarizer. We confirm that the polarization state of light remains practically unchanged after propagating through the samples used in this investigation. Light only loses its polarization state in samples that are multiple times thicker than the transport mean free path $l_t = l_s/(1-g)$, ($l_t >> l_s$ for most biological tissues) (*46*).

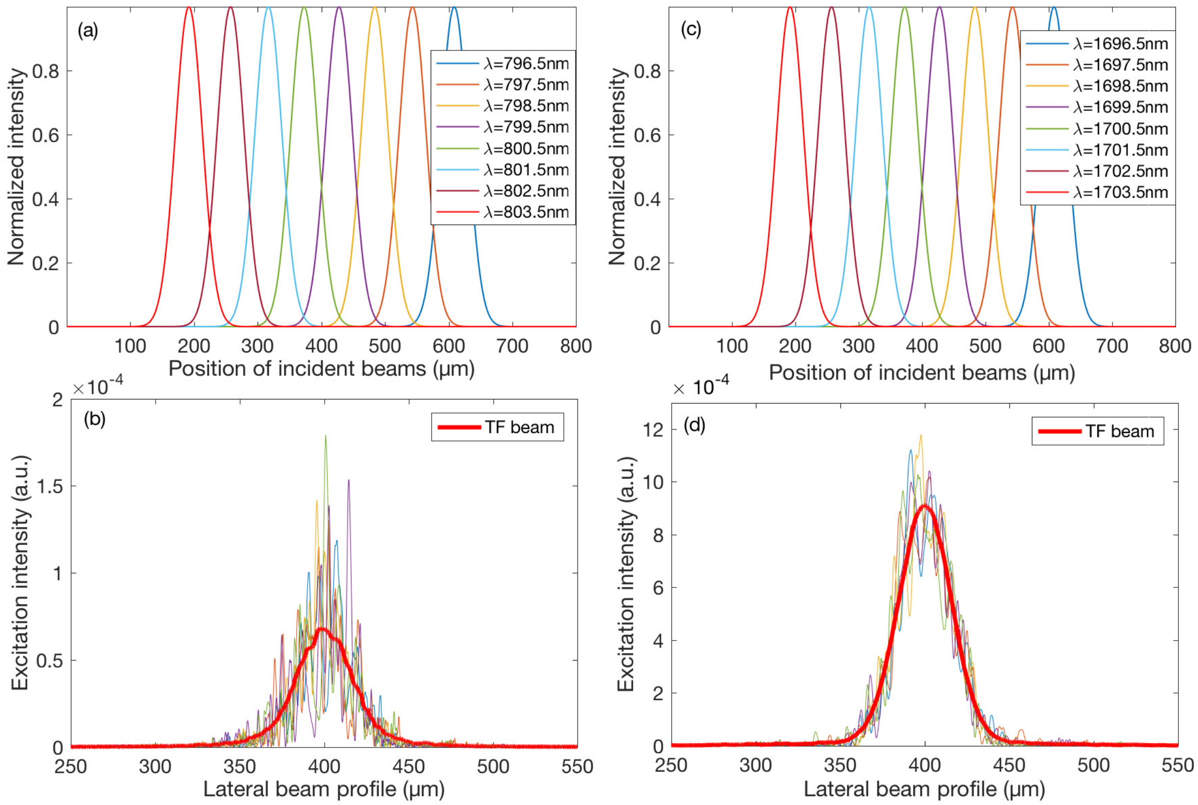

Fig. S1. **Numerically simulated TF laser beam propagating through 400 μm of brain tissue.** (**a**) The laser pulse, having central wavelength at 800 nm and (**c**) central wavelength at 1700 nm, is dispersed into its constituent frequencies, each represented as a Gaussian beam (FWHM = 35 μm), at the tissue surface. (**b**, **d**) Beam profiles at the focal plane for two- and three-photon excitation, respectively. The solid red curve represents the overall lateral beam profile after propagating through brain tissue showing how speckle pattern is considerably smoothed out.

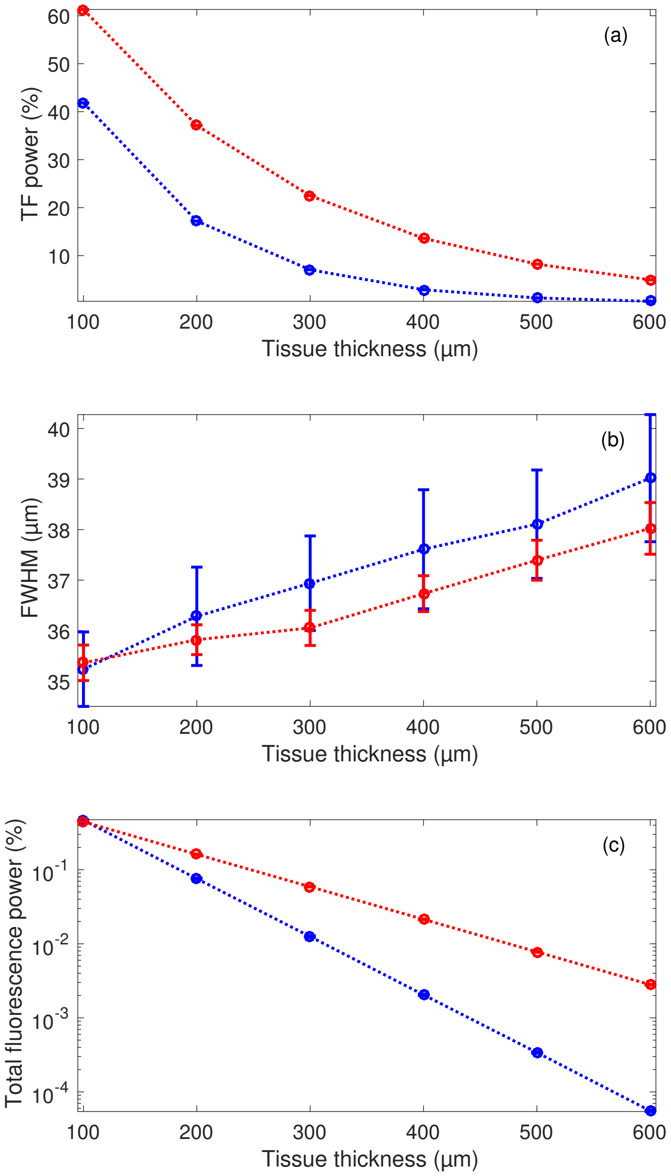

Fig. S2. **Properties of a numerically simulated TF laser beam through brain tissue. (a)** Total TF laser power at focal plane, **(b)** FWHM of a TF Gaussian beam and **(c)** total collected fluorescence power using a NA = 0.8 objective, for different thicknesses of brain tissue. Incident laser power is set to 100 (a.u.). Blue and red curves correspond to simulated two- and three-photon fluorescence excitation, respectively.

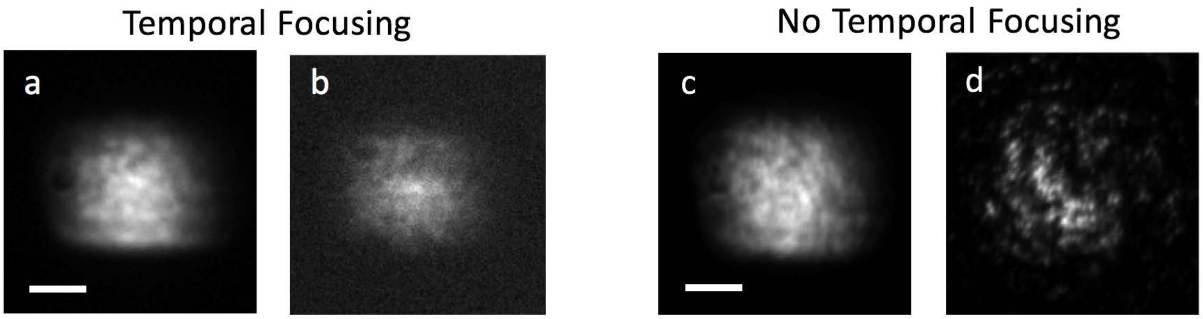

Fig. S3. **Effect of scattering on the beam profile with and without TF.** (**a**, **b**) Beam profile with and (**c**, **d**) without TF. (**a**, **c**) were taken without scattering and (**b**, **d**) through 900 µm of scattering phantom ($l_s \approx 250\,\mu\text{m}$). Scale bar is 20 µm.

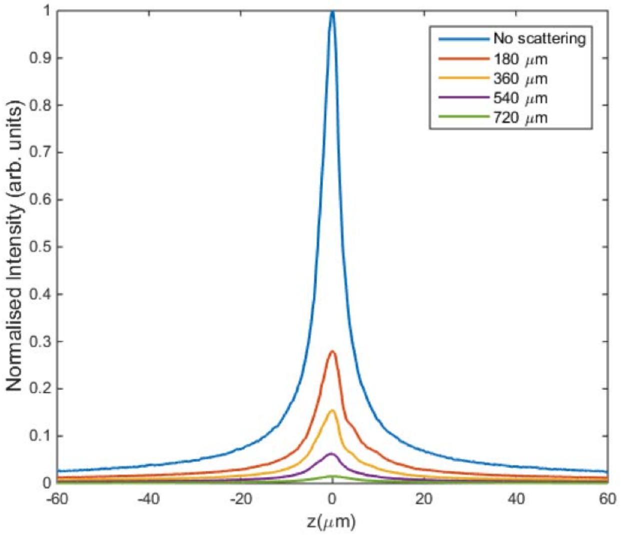

Fig. S4. **Depth profile of a TF beam through a scattering phantom.** The axial extent of the illumination plane was measured through different thicknesses of scattering phantom ($l_s \approx 250\,\mu\text{m}$). The size at the FWHM determines the depth resolution of the microscope. The axial resolution without scattering is 4.7 ± 0.5 µm. Objective used for this measurement: Nikon $40\times$ NA = 0.8.

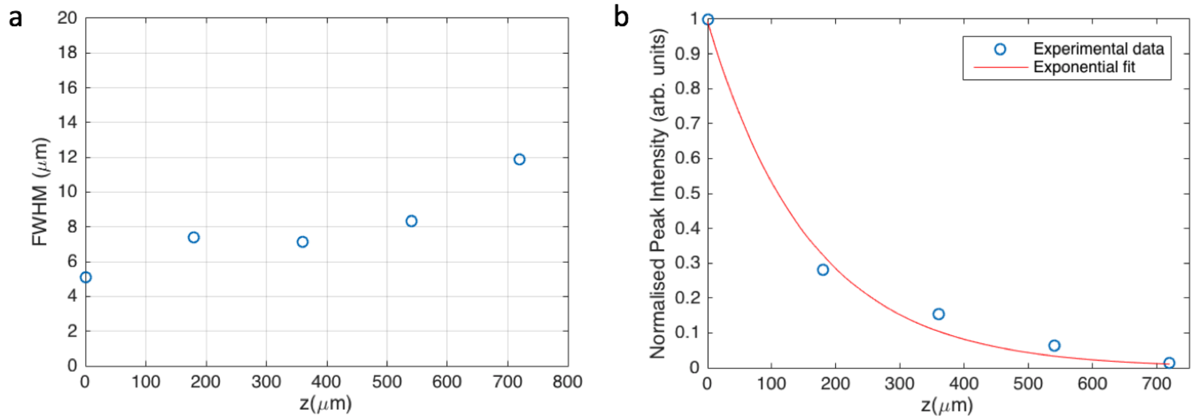

Fig. S5. **Characterization of a TF beam through a scattering phantom.** Plots obtained from the data represented in fig. S4. (**a**) Full width at half maximum (FWHM) of the depth profiles. The depth resolution decreases about 2.5 times after propagating through 720 μm of the scattering phantom ($l_s \approx 250\,\mu\mathrm{m}$). (**b**) Peak intensity of the depth profiles. It decreases exponentially as light travels farther through the scattering medium.

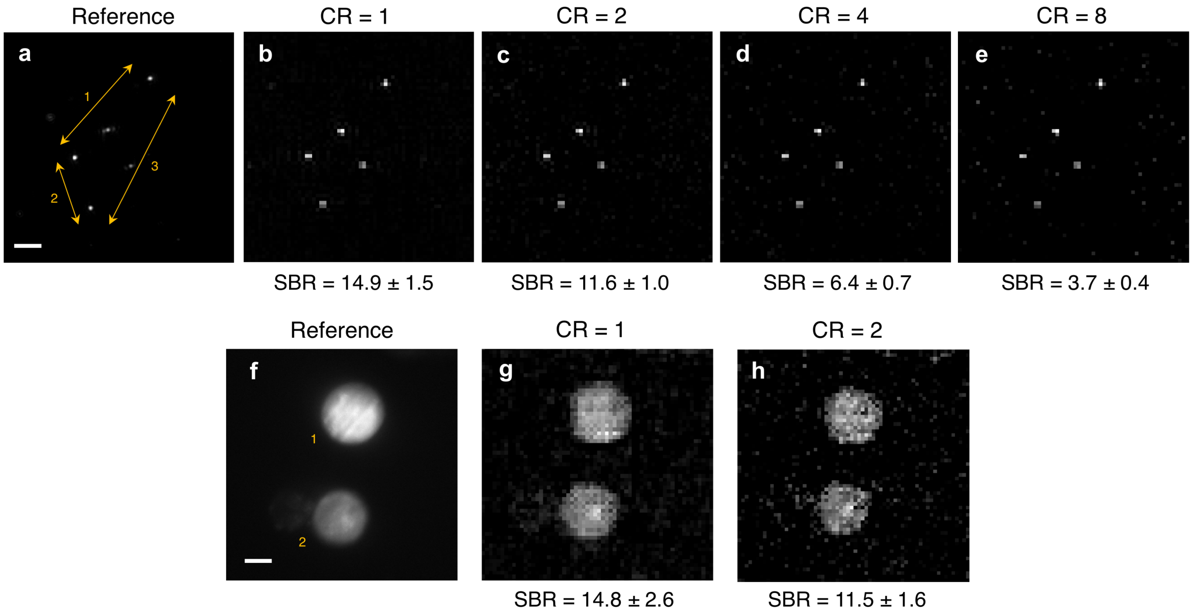

Fig. S6. **Images of fluorescent microscopic samples without scattering.** Fluorescent beads of 400 nm in diameter and fixed HEK293T/17-GFP cells were imaged without the presence of any scattering layer. (**a**, **f**) Images taken from the reference imaging arm under uniform TF illumination across the FOV. No camera binning was used. Exposure time was 5 s and 10 s, respectively. (**b-e**, **g-h**) Images obtained in epifluorescence configuration with TRAFIX using a Hadamard basis containing 4096 illumination patterns. They were reconstructed using different compression ratios corresponding to 100% (CR = 1), 50% (CR = 2), 25% (CR = 4) or 12.5% (CR = 8) of the total patterns. Each single measurement of the Hadamard scan was taken with a binning of 64×64 and an exposure time of 0.025 s and 0.02 s, respectively. The spacing between beads (table S3) and the diameter of the cells (table S2) were measured to assess image quality. The SBR is shown for all reconstructed images. Scale bars are 10 μm.

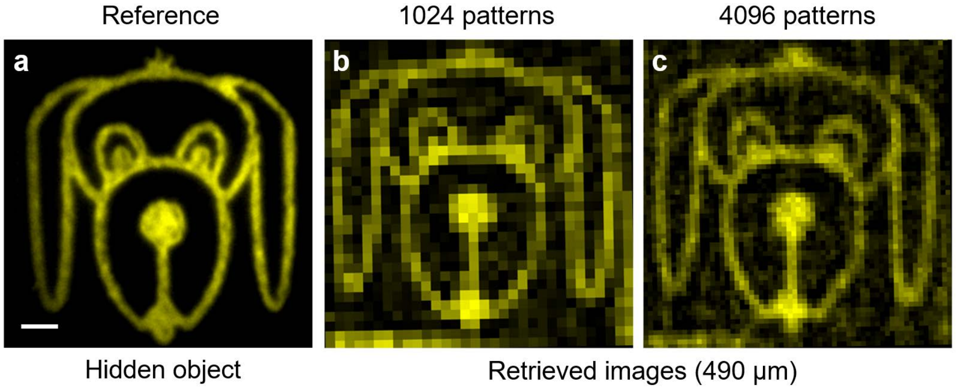

Fig. S7. **Comparison of a hidden object and retrieved images through a scattering phantom with different resolution.** (**a**) Reference image of the fluorescent micropattern without any scattering sample. (**b**, **c**) Reconstructed images obtained with TRAFIX through 490 μm of scattering phantom. The two retrieved images were reconstructed using full Hadamard bases containing 1024 and 4096 patterns, respectively. Scale bar is 10 μm.

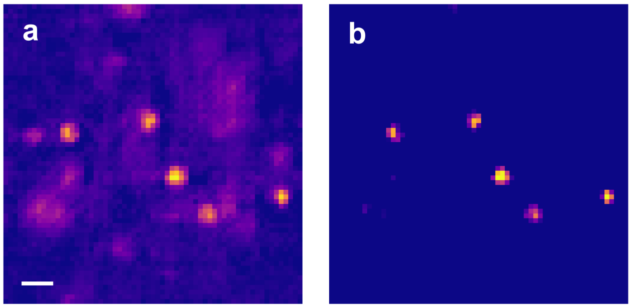

Fig. S8. **Image of 4.8-μm fluorescent beads in a volumetric scattering phantom.** 0.4 μm fluorescent beads were embedded in the phantom to generate additional background fluorescent light. (a) Image taken at a depth of ∼ 300 μm with balanced gray levels. (b) Same image with gray levels adjusted to minimize background noise. Scale bar is 10 μm

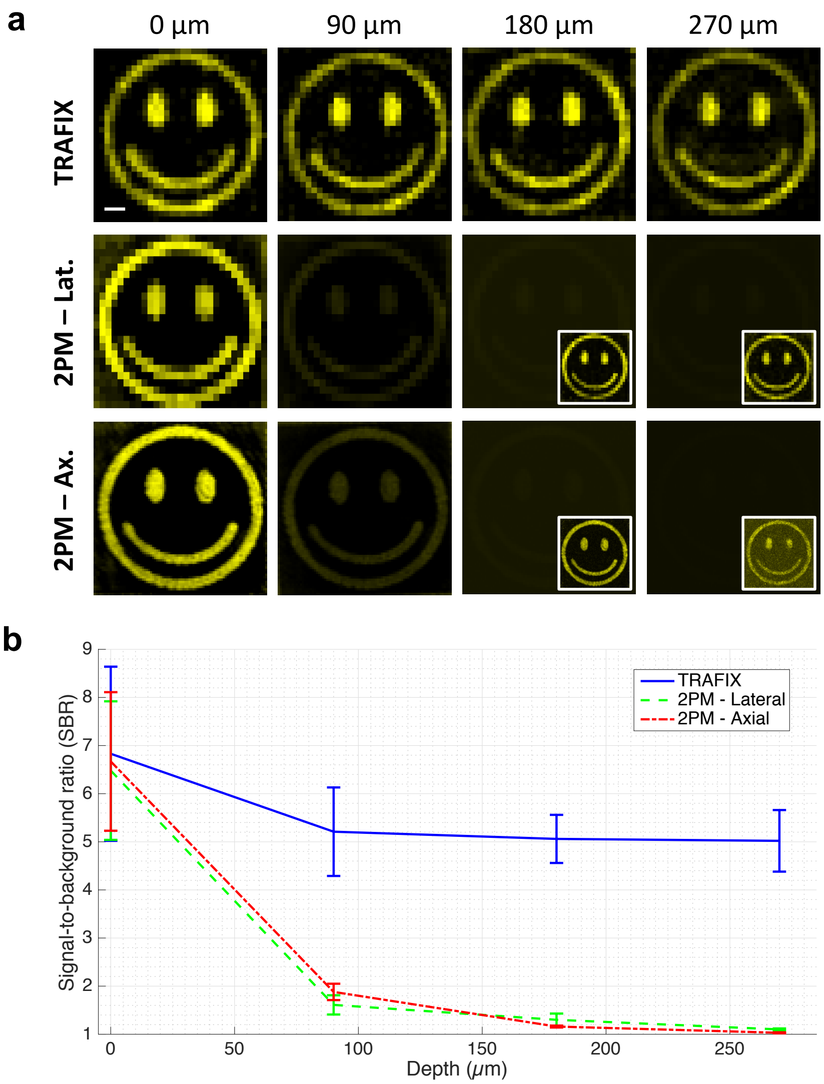

Fig. S9. **Comparison of SBR of TRAFIX and point-scanning two-photon microscopy (2PM) at depth.** **(a)** Images of fluorescent micropatterns obtained with TRAFIX and 2PM through scattering phantoms of different thicknesses. 2PM - Lateral has approximately the same lateral resolution as TRAFIX and 2PM-Axial has similar axial confinement as TRAFIX. Small insets show the same image with different gray values. Laser power per unit area per TRAFIX pattern was 0.068 mW/ μm^2·pattern and the total accumulated laser power per unit area after a full measurement with 1024 patterns was 69 mW/ μm^2. Laser power per unit area for 2PM was 2.2 mW/ μm^2. Camera binning and exposure time were maintained constant for all measurements at each depth. Exposure time per TRAFIX pattern was the same as the pixel dwell time in 2PM. Images were acquired with an underfilled 20× NA = 0.75 objective. Scale bar is 10 µm. **(b)** Values of SBR at different depths for TRAFIX and 2PM. The scattering mean free path of the phantom is $l_s \approx 100$ µm.

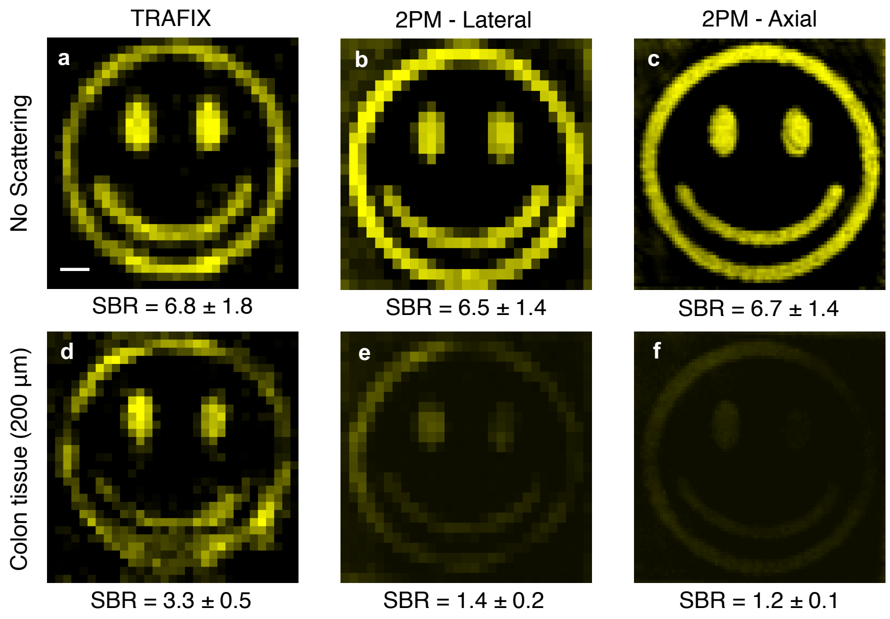

Fig. S10. **Comparison of TRAFIX and point-scanning two-photon microscopy (2PM) through human colon tissue.** **(a-c)** Images of a fluorescent micropattern obtained with TRAFIX and 2PM without scattering. 2PM-Lateral has approximately the same lateral resolution as TRAFIX and 2PM-Axial has similar axial confinement as TRAFIX. **(d-f)** Images obtained through 200 μm of unfixed human colon tissue. Laser power per unit area per TRAFIX pattern was 0.068 mW/ μm^2·pattern and the total accumulated laser power per unit area after a full measurement with 1024 patterns was 69 mW/ μm^2. Laser power per unit area for 2PM was 2.2 mW/ μm^2. Camera binning and exposure time were maintained constant for **(a-c)** and **(d-f)**. Exposure time per TRAFIX pattern was the same as the pixel dwell time in 2PM. SBR is shown for all images. Images were taken with an underfilled 20× NA = 0.75 objective. Scale bar is 10 μm.

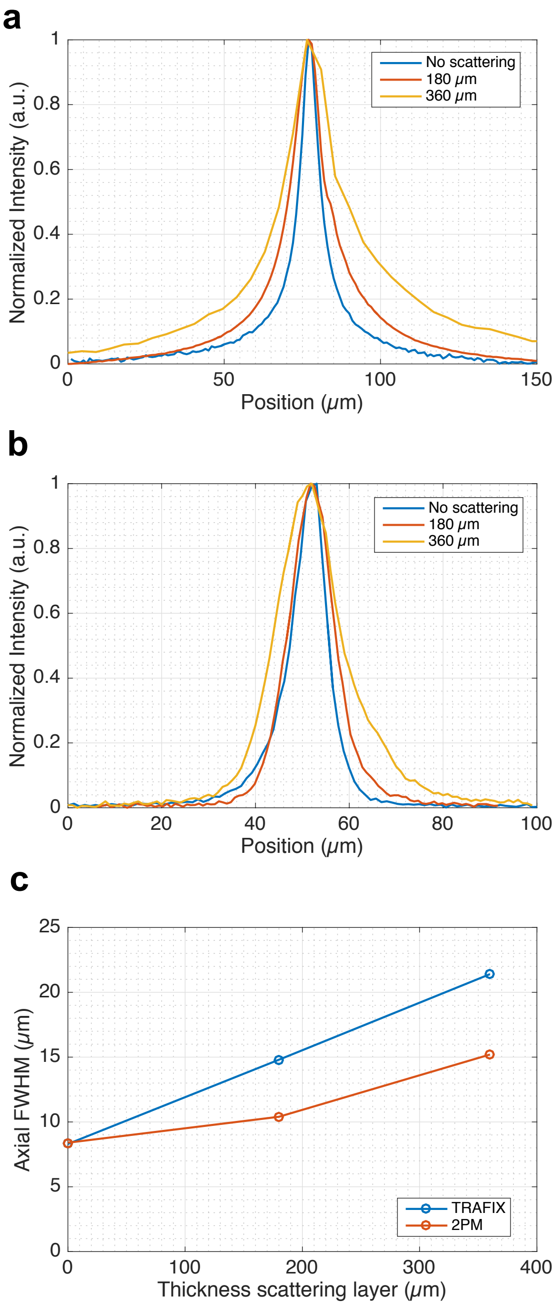

Fig. S11. **Axial confinement in TRAFIX and point-scanning two-photon microscopy (2PM).** Axial profile of **(a)** a uniform TF 84 μm by 84 μm square and **(b)** a spatially focused 1.26 μm diameter spot through various layers of scattering phantom. $l_s \approx 150\,\mu\mathrm{m}$ **(c)** Full width at half maximum (FWHM) of the depth profile for TRAFIX and 2PM through scattering phantoms. Objective used in this experiment: Nikon 20× NA = 0.75.

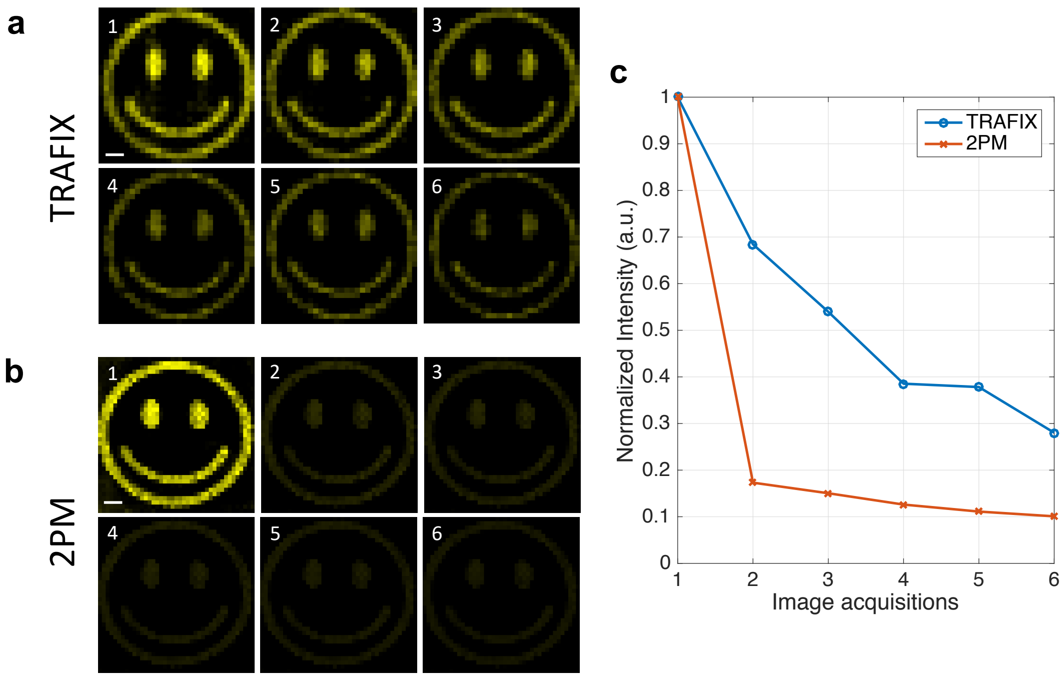

Fig. S12. **Photobleaching comparison of TRAFIX and point-scanning two-photon microscopy (2PM).** Successive images obtained with **(a)** TRAFIX and with **(b)** 2PM without scattering. Laser power per unit area for TRAFIX is 0.034 mW/ μm^2·pattern and the resulting accumulated power per unit area after projecting 1024 patterns is 35 mW/ μm^2. The laser power per unit area for 2PM is 0.55 mW/ μm^2, which corresponds to half of the required power per unit area to generate the same total fluorescence signal as TRAFIX. Images taken with an underfilled 20× NA = 0.75 objective. **(c)** Fluorescence intensity decay after several acquisitions for TRAFIX and 2PM.

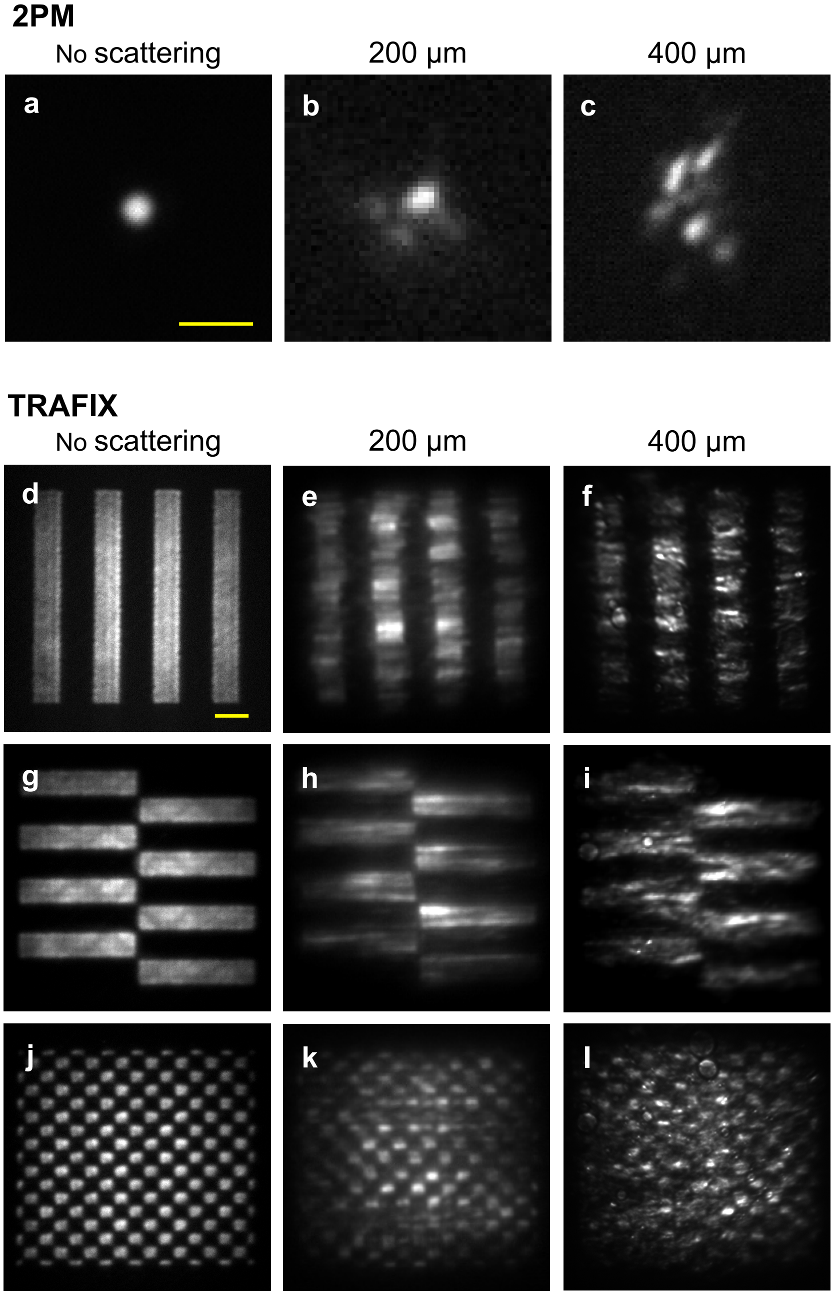

Fig. S13. **Effect of scattering on illumination beams in point-scanning two-photon microscopy (2PM) and TRAFIX.** The three columns correspond to the illumination patterns without scattering, through 200 µm and 400 µm of unfixed colon tissue, respectively. (**a**-**c**) 4 µm diameter focused beam in 2PM. (**d** -**l**) Hadamard patterns of different orientation and spatial frequencies in TRAFIX. The size of the patterns is 130 µm by 130 µm. Scale bars are10 µm in (**a**) and 20 µm in (**d**). This experiment was peformed with an underfilled of 40× NA = 0.8 objective.

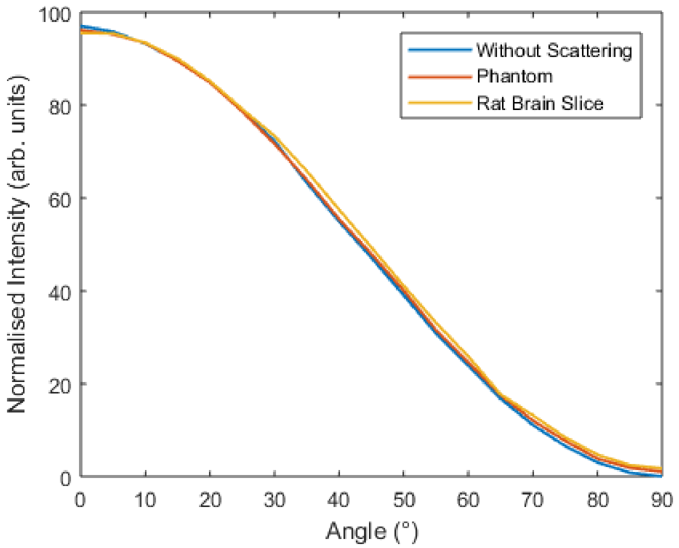

Fig. S14. **Effect of turbid media on light polarization.** Linearly polarized illumination light retains its polarization state through propagation in the scattering phantom or rat brain tissue used in this experiment. The data clearly obeys Malus' Law. The thickness of the scattering phantom and the rat brain tissue were 640 µm and 400 µm, respectively.

Table S1. **SBR measured for images shown in this work.** Numerical columns correspond to retrieved images obtained through scattering media with TRAFIX at different CRs.

	**SIGNAL-TO-BACKGROUND RATIO**			
	CS (CR=1)	CS (CR=2)	CS (CR=4)	CS (CR=8)
Beads Without Scattering	14.9 ± 1.5	11.6 ± 1.0	6.4 ± 0.7	3.7 ± 0.4
Beads Phantom	5.5 ± 2.1	4.2 ± 1.8	3.9 ± 1.5	2.6 ± 0.7
Beads Colon	5.4 ± 1.0	–	–	–
Cells Without Scattering	14.8 ± 2.6	11.5 ± 1.6	–	–
Cells Phantom	4.9 ± 1.5	4.3 ± 0.4	–	–
Cells Colon	4.6 ± 0.3	–	–	–

Table S2. **Cell diameters of all images shown in this work.** Cells correspond, in order, to fig. S6, Fig. 2 and Fig. 3. The deviation from the reference value is shown in the last two columns.

	Cell	Diameter FWHM (μm)			Deviation (%)	
		**Reference**	**CR=1**	**CR=2**	**CR=1**	**CR=2**
Without scattering	1	19.20	18.92	20.44	1.5	6.4
	2	16.31	16.35	14.73	0.2	9.7
Scattering phantom	1	20.66	20.64	19.23	0.1	6.9
	2	14.32	13.86	12.71	3.3	11.2
Colon tissue	1	13.07	13.91	–	6.5	–
	2	13.94	11.71	–	16.0	–
	3	13.22	12.07	–	8.6	–
	4	12.22	13.32	–	9.1	–
	5	11.65	12.53	–	7.6	–

Table S3. **Beads spacing corresponding to all images shown in this work.** They refer, in order, to fig. S6, Fig. 2 and Fig. 3. The deviation from the reference value is shown in the last four columns.

	**No.**	**Distance (μm)**					**Deviation (%)**			
		**Ref.**	**CR=1**	**CR=2**	**CR=4**	**CR=8**	**CR=1**	**CR=2**	**CR=4**	**CR=8**
Without scattering	1	42.19	40.99	40.29	41.46	40.38	2.8	4.5	1.7	4.2
	2	20.40	19.16	19.81	19.73	19.54	6.1	2.9	3.3	4.3
	3	55.25	52.95	52.15	54.02	52.88	4.2	5.6	2.2	4.2
Scattering phantom	1	23.21	23.07	23.05	23.51	23.57	0.6	0.7	1.3	1.6
	2	18.36	17.87	18.04	18.19	18.80	2.7	1.7	0.9	2.4
	3	39.90	40.04	40.04	39.37	39.69	0.4	0.4	1.3	0.5
Colon tissue	1	13.38	15.39	-	-	-	15.0	-	-	-
	2	38.31	44.55	-	-	-	16.3	-	-	-
	3	30.77	35.88	-	-	-	16.0	-	-	-
